# Integrated transcriptomics and metabolomics analyses provide new insights into cassava in response to nitrogen deficiency

**DOI:** 10.3389/fpls.2024.1488281

**Published:** 2025-01-14

**Authors:** Yu Wang, Jing Chu, Haoyang Zhang, Hao Ju, Qing Xie, Xingyu Jiang

**Affiliations:** ^1^ National Center of Technology Innovation for Saline-Alkali Tolerant Rice, College of Coastal Agricultural Sciences, Guangdong Ocean University, Zhanjiang, China; ^2^ Hainan Key Laboratory for Biotechnology of Salt Tolerant Crops/Institute of Tropical Crops, Hainan University, Haikou, China

**Keywords:** transcriptomics, metabolomics, nitrogen deficiency, flavonoid biosynthesis, cassava

## Abstract

Nitrogen deficiency is a key constraint on crop yield. Cassava, the world’s sixth-largest food crop and a crucial source of feed and industrial materials, can thrive in marginal soils, yet its yield is still significantly affected by limited nitrogen availability. Investigating cassava’s response mechanisms to nitrogen scarcity is therefore essential for advancing molecular breeding and identifying nitrogen-efficient varieties. This research undertook a comprehensive analysis of cassava seedlings’ physiological, gene expression, and metabolite responses under low nitrogen stress. Findings revealed that nitrogen deficiency drastically suppressed seedling growth, significantly reduced nitrate and ammonium transport to aerial parts, and led to a marked increase in carbohydrate, reactive oxygen species, and ammonium ion levels in the leaves. Transcriptomic and metabolomic analyses further demonstrated notable alterations in genes and metabolites linked to carbon and nitrogen metabolism, flavonoid biosynthesis, and the purine metabolic pathway. Additionally, several transcription factors associated with cassava flavonoid biosynthesis under nitrogen-deficient conditions were identified. Overall, this study offers fresh insights and valuable genetic resources for unraveling cassava’s adaptive mechanisms to nitrogen deprivation.

## 1 Introduction

Nitrogen is an essential macronutrient critical for plant growth and development, forming a fundamental component of cellular constituents like nucleic acids, proteins, and chlorophyll (Luo et al., 2020). Plants absorb nitrogen from the soil via extensive root systems, and soil nitrogen levels significantly influence primary and secondary metabolism, ultimately determining plant yield and quality (Cao et al., 2019). In natural settings, plants commonly experience nitrogen-limited environments, which substantially challenge to crop productivity (Tabata et al., 2014). Consequently, agricultural practices often rely on substantial nitrogen fertilizer inputs to enhance crop yield and quality. However, over 50% of the applied nitrogen escapes into the environment rather than being absorbed by plants, leading to severe environmental pollution and increasing modern agricultural costs (Ahmed et al., 2017). Thus, understanding the molecular mechanisms behind plant adaptation to low nitrogen, and developing varieties with enhanced nitrogen uptake and utilization efficiency are critical. These advancements could help maintain or improve crop quality and yield while reducing nitrogen fertilizer usage.

The impact of nitrogen scarcity on plants has been widely studied, showing that limited nitrogen generally inhibits plant growth, reduces photosynthetic rates, and disrupts nitrogen absorption as well as assimilation (Mu and Chen, 2021). As immobile organisms, plants cannot relocate to more favorable conditions therefore, they evolved complex adaptive strategies to cope with nutrient stress. When soil nitrogen levels decrease, plants adjust their root architecture—such as elongating the main root and developing root hairs to improve nitrogen acquisition (López-Bucio, 2003). This adaptation is critical for plant survival under low nitrogen conditions, as evidenced by the significant increase in root-to-shoot ratios in rice (Nada et al., 2019), the marked enhancement of nitrogen acquisition through root hair formation (Jia et al., 2023). Additionally, accelerated leaf senescence serves as an important adaptive strategy, where biomolecules like proteins, nucleic acids, and lipids are degraded, allowing nitrogen to be relocated from aging leaves to younger tissues, thus supporting continued growth and reproduction. This process of nitrogen remobilization and redistribution is particularly pronounced under nitrogen stress (Agüera et al., 2010). In response to low nitrogen conditions, plants modulate the expression of specific genes through the accumulation of secondary metabolites. Genes and metabolites associated with phenylpropanoids and their downstream pathways have been identified as key players in this adaptation. For instance, two genes encoding Phenylalanine ammonia-lyase (PAL) in *Arabidopsis*, *AtPAL1* and *AtPAL2*, were upregulated in low nitrogen conditions, resulting in increased levels of metabolites such as quercetin and kaempferol (Olsen et al., 2008). Additionally, low nitrogen significantly enhances the accumulation of flavonoid in *Arabidopsis* (Stewart et al., 2001), and transient nitrogen deficiency raises chlorogenic acid and rutin concentrations in tomato leaves (Bénard et al., 2011). Autophagy proteins (ATG) are also involved in the synthesis of plant flavonoids under low nitrogen conditions, overexpression of *MdATG18a* in Arabidopsis and apple leads to anthocyanin accumulation, thereby improving nitrogen depletion tolerance (Sun et al., 2018). Furthermore, specific transcription factors are pivotal in plant responses to low nitrogen stress. overexpression of *SiMYB30* enhances the tolerance of transgenic rice to low nitrogen stress (Zhang et al., 2023). The apple miR164-*MhNAC1* module modulates nitrogen absorption by influencing citric acid secretion from roots (Wang et al., 2024). In Arabidopsis, *BES1* facilitates nitrate uptake under low nitrogen conditions by directly regulates the expression level of genes related to nitrogen uptake (Wang et al., 2023a). Despite these findings, the metabolic pathways and regulatory mechanisms governing plant responses to nitrogen deficiency remain largely unexplored, with potential variations in adaptation mechanisms across different plant species.

Cassava (*Manihot esculenta* Crantz), a perennial shrub belonging to the Euphorbiaceae family (Blagbrough et al., 2010), is valued for its starch-rich tuberous roots, serving as both a staple food crop and a key raw material for industrial processing. Additionally, its stems and leaves are utilized in papermaking, alcohol production, and animal feed (Li et al., 2017). Known for its ability to thrive in infertile soils, cassava exhibits strong resilience to nitrogen deprivation (Xu et al., 2013). However, declining soil fertility and limited fertilizer resources have constrained its yield, nitrogen deficiency has been identified as the main cause of cassava’s low productivity, which affects stay green ability, photosynthetic rate, translocation of organic compounds and tuberous root yield (Tafur et al., 1998; De Souza et al., 2017), However, cassava plants grown in low-nitrogen soils generally have the highest nitrogen use efficiency (Cruz et al., 2003). Therefore, understanding cassava’s response mechanisms to nitrogen deficiency is essential for cultivating high nitrogen use efficiency (NUE) varieties whiles enhancing yield. Despite its importance, research on cassava’s nitrogen nutrition is limited, with the physiological and molecular mechanisms underlying its response to low nitrogen stress remaining unclear. This study examines cassava’s leaf and it root responses to nitrogen deficiency through physiological, transcriptomic, and metabolomic analyses. The findings will provide valuable insights into the genes and metabolites involved in cassava’s tolerance to low nitrogen, offering a theoretical foundation for enhancing nitrogen utilization in cassava crop and lowering fertilizer use.

## 2 Materials and methods

### 2.1 Plant materials

The experiment was carried out at Guangdong Ocean University (Zhanjiang, Guangdong) using cassava variety SC8. SC8 tissue culture seedlings were initially transferred to a substrate composed of an equal mixture of peat soil, vermiculite and grown in a temperature-controlled greenhouse under natural light until reaching approximately 40 cm in height. At this stage, stem segments containing a single bud were excised, sterilized with 75% ethanol and 10% sodium hypochlorite, and transferred to an MS medium. These explants were cultured in a 26°C light incubator until rooting, after which they were moved to a hydroponic system containing Afdonin nutrient solution within a temperature-controlled greenhouse. One week later, seedlings of uniform size were placed into a continuously aerated Afdonin nutrient solution for hydroponic growth. The composition of the nutrient solution included NaH_2_PO_4_·H_2_O 0.1g/L; Na_2_HPO_4_·12H_2_O 0.1g/L; KCl 0.15g/L; CaCl_2_·2H_2_O 0.36g/L; MgSO_4_·7H_2_O 0.5g/L; EDTA-Fe 0.0025mg/L; H_3_BO_3_ 2.86mg/L; CuSO_4_·5H_2_O 0.08mg/L; ZnSO_4_·7H_2_O 0.22mg/L; MnCl_2_·4H_2_O 1.81mg/L; and NaMoO_4_ · 4H_2_O 0.09 mg/L. For the nitrogen treatments, normal nitrogen (NN) and low nitrogen (LN) conditions were established by adding 3 mM and 0.3 mM NH_4_NO_3_ respectively as the sole nitrogen source. After one week of cultivation, roots and the top 3-4 leaves from different treatments were collected and rapidly frozen in liquid nitrogen for subsequent metabolite analysis, RNA extraction, and physiological measurements.

### 2.2 Measurement of plant height, biomass and root morphometry

The height of the plants was measured using a ruler. Hydroponic plants were carefully removed from the culture vessels and excess water on the plant surfaces was gently blotted using absorbent paper. The fresh weight of the aerial parts and roots was then measured with an electronic balance accurate to 0.01 g. Root morphology analyzed using the Scanmarker i800 Plus (Microtek Co., Ltd., China).

### 2.3 Determination of the SPAD value and N content

The SPAD value and nitrogen content in the leaves were determined using the TYS-4N analyzer (TOP Cloud-agri Technology Co., Ltd., China). For the collection of xylem sap, the stems were cut off with a blade at a distance of 1 cm above the root at dark conditions, and fluid exuding from the root was collected (Alvarez et al., 2008). Nitrate and ammonium content of different samples were quantified according to the protocols provided by the respective kits (G0410F, G0409F) produced by Suzhou Grace Biotechnology Co., Ltd. (Suzhou, China). In short, 0.1g frozen leaf and root samples were homogenized in the extraction solution provided by the kit at 4°C and centrifuged at 12,000 rpm at 4°C for 10 minutes, the supernatant were used for measurement, the xylem sap was directly detected without treatment. The OD values were determined at 410 and 570 nm, then, the content of nitrate and ammonium of each sample were calculated according to the manufacturer’s instructions. All the measurements in this part were completed with three biological replicates.

### 2.4 Determination of physiological resistance indices

For enzymatic and biochemical analyses, 0.1 g of fresh plant tissue was weighed and homogenized in 1 ml of extraction buffer (0.05 M phosphate buffer, pH 7.8) using a freezing grinder. The homogenates were centrifuged at 12,000 rpm for 20 minutes at 4°C, and the resulting supernatant was used as the enzyme source. The content of malondialdehyde (MDA) was determined using the thiobarbituric acid (TBA) method (Kosugi and Kikugawa, 1985). Soluble protein content was assessed by Coomassie Brilliant Blue (CBB) colorimetry (Bradford, 1976). superoxide dismutase (SOD) activity was measured by the riboflavin-NBT method (Beauchamp and Fridovich, 1971), specifically, 3 mL reaction mixture was prepared for each sample, consisting of 25 mM potassium phosphate buffer (pH=7.8), 13 mM methionine, 75 μM NBT, 2 μM riboflavin, 100 uM EDTA, and 20 μL enzyme extract. The reaction mixture was irradiated at a light intensity of 4000 lx for 30 minutes, and then the absorption value of the reaction solution at 560 nm was determined. with one unit of SOD defined as the amount of enzyme inhibiting 50% of NBT photoreduction. peroxidase (POD) activity was evaluated using the guaiacol method (Li et al., 2013), specifically, 3ml reaction mixture was prepared for each sample. The reaction solution consisted of 100uM potassium phosphate buffer, guaiacol and 30% H_2_O_2_ with a volume ratio of 5000:28:19. Then 20ul enzyme solution was added to the reaction solution, quickly mixed and measured its light absorption value at 470nm. The value was recorded every 30s for 5 times, with one enzyme unit defined as the amount of enzyme increasing absorbance by 0.01 in 1 min. All the measurements in this part were completed with three biological replicates.

### 2.5 Metabolite extraction and LC/MS analysis

The four sample groups were designated as follows: LL (leaves under low nitrogen conditions), NL (leaves under normal nitrogen conditions), LR (roots under low nitrogen conditions), and NR (roots under normal nitrogen conditions). Each group comprised six biological replicates, with each replicate consisting of four individual seedlings. Tissue samples (100 mg) were individually ground in liquid nitrogen, and the resulting homogenate was resuspended in prechilled 80% methanol, followed by thorough vortexing. The samples were then incubated on ice for 5 minutes and centrifuged at 15,000 g at 4°C for 20 minutes. A portion of the supernatant was diluted with LC-MS grade water to achieve a final methanol concentration of 53%. These diluted samples were transferred to fresh Eppendorf tubes and subjected to another centrifugation at 15,000 g at 4°C for 20 minutes. The supernatant was then injected into the LC-MS/MS system for analysis (Thermo Fisher, Q Exactive™ HF/Q Exactive™ HF-X, Germany; Thermo Fisher, Vanquish UHPLC, Germany).

Mass spectrometry data were processed using CD 3.3 software, screening for parameters such as retention time and mass-to-charge ratio for each metabolite. The data were compared with the mzCloud (https://www.mzcloud.org/), mzVault, and Masslist databases, leading to the identification and relative quantification of metabolites. Identified metabolites were further annotated using the Kyoto Encyclopedia of Genes and Genomes (KEGG) database (https://www.genome.jp/kegg/pathway.html), human metabolome database (HMDB) database (https://hmdb.ca/metabolites), and the LIPID Maps database (http://www.lipidmaps.org/). MetaX software was employed for principal component analysis (PCA), partial least squares discriminant analysis (PLS-DA), and the calculation of fold changes (FC) in metabolites between groups. Metabolites were classified as differential if they met the criteria of VIP > 1, P value < 0.05, and FC ≥ 2 or FC ≤ 0.5 between comparison groups. Finally, the KEGG database was utilized to conduct enrichment analysis of the differential metabolites.

### 2.6 Total RNA isolation, RNA-Seq library construction, and Illumina sequencing

The duplicates of each pair of metabolome samples were combined in equal amounts, and total RNA was extracted using the RNA extraction kit (Tiangen, China, DP437). The quality of the RNA was assessed with an Agilent 2100 bioanalyzer, and qualified samples were then sent to Novogene Co. for library construction and sequencing. The raw sequencing data were filtered, and the resulting clean reads were mapped to the cassava reference genome (Bredeson et al., 2016) (https://www.ncbi.nlm.nih.gov/datasets/genome/GCF_001659605.2/) using HISAT2. FeatureCounts (1.5.0-p3) (Liao et al., 2014) was employed to calculate FPKM values (fragments per kilobase of transcript per million mapped reads) for each transcript. Differential expression analysis between comparison groups was performed using DESeq2 software (1.20.0) (Love et al., 2014), with genes exhibiting |log2(Fold Change)| ≥ 1 and padj ≤ 0.05 considered differentially expressed. ClusterProfiler (3.8.1) was then utilized for Gene Ontology (GO) and KEGG (Ogata et al., 1999) enrichment analysis of the differentially expressed genes.

### 2.7 Co-expression network analysis

Pearson correlation coefficients (PCCs) were calculated based on the FC values of the differentially expressed genes (DEGs), and only correlations with absolute PCC values exceeding 0.8 were included in the analysis. The resulting co-expression network was visualized using Cytoscape software (version 3.6.1).

### 2.8 Quantitative real-time PCR validation

For qRT-PCR analysis, 1 µg of RNA was reverse transcribed into cDNA using MightyScript Plus First Strand cDNA Synthesis Master Mix (Sangon, China, B639252), which served as the template for the qRT-PCR reactions. These reactions were conducted on a CFX96 Touch instrument (Bio-Rad, USA) using SGExcel FastSYBR Mixture (Sangon Biotech, China, B532955). The reaction conditions were as follows: 95°C for 3 minutes (initial hold), 95°C for 5 seconds, and 60°C for 20 seconds (PCR stage, 40 cycles). This was then followed by a melt curve analysis according to standard protocols. *MeUbq9* was used as the reference gene (Irigoyen et al., 2020; Zhu et al., 2022), and the 2^−△△Ct^ method was employed to determine the relative expression levels of the genes. Primer sequences for all selected genes are provided in **Supplementary Table S1**, and each sample was analyzed in triplicate.

### 2.9 Statistical analysis

Experimental data were processed using Microsoft Excel 2010 and are expressed as mean ± SE. Student’s t-test was performed using GraphPad Prism software, significance of differences is shown in figures as ** P < 0.01. Each experiment included a minimum of three replicates.

## 3 Results

### 3.1 The growth, photosynthesis, and nitrogen accumulation of cassava seedlings

**Figure 1:**
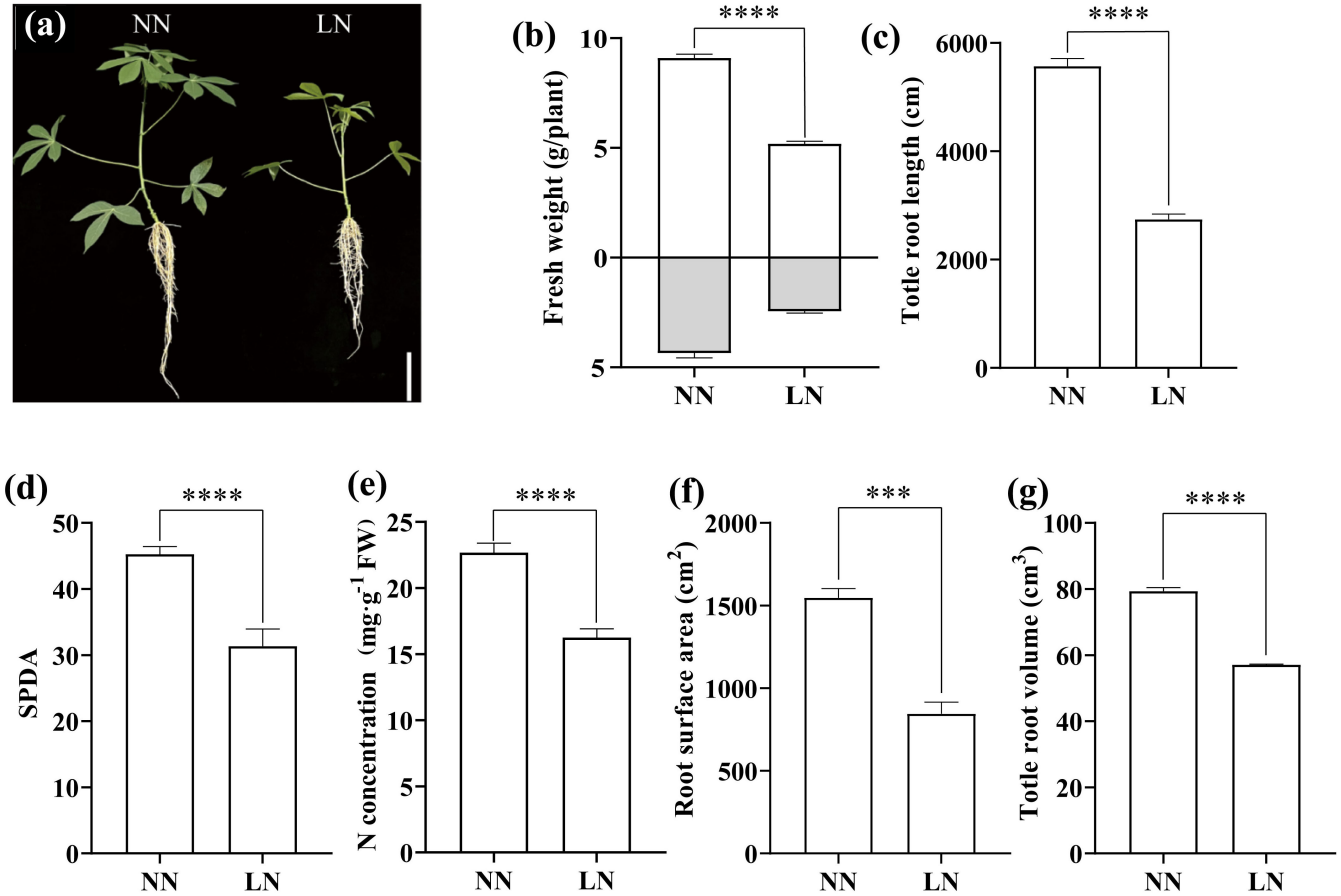
Effects of Low Nitrogen on Cassava Growth, Chlorophyll, and Nitrogen Content. **(A)** Phenotype of cassava seedlings. **(B)** Fresh weight. **(C)** Total root length. **(D)** Relative chlorophyll content (SPAD). **(E)** Total nitrogen content. **(F)** Root surface area. **(G)** Total root volume. Twelve-day-old seedlings were transferred to low nitrogen (LN) and normal nitrogen (NN) treatments for one week. Data are presented as means ± SD (n ≥ 3). *** means p ≤0.001 and **** means p ≤0.0001.

Under hydroponic conditions, low nitrogen (LN) treatment significantly altered the morphology of cassava seedlings, primarily evidenced by reduced plant height, root length (**Figure 1A**), and biomass accumulation (**Figure 1B**). Root scanning further revealed that, the total root length, root area, and total root volume in the LN-treated seedlings were markedly lower compared to those in the NN group (**Figures 1C, F, G**). Additionally, LN treatment caused severe leaf yellowing, with the SPAD value decreasing by 30.7% relative to the NN group (**Figure 1D**). The LN treatment also impeded nitrogen accumulation in the leaves, with nitrogen content in the LN group reaching only 71.7% of that in the NN group (**Figure 1E**). These results suggest that inadequate nitrogen supply severely hampers cassava growth.

**Figure 2:**
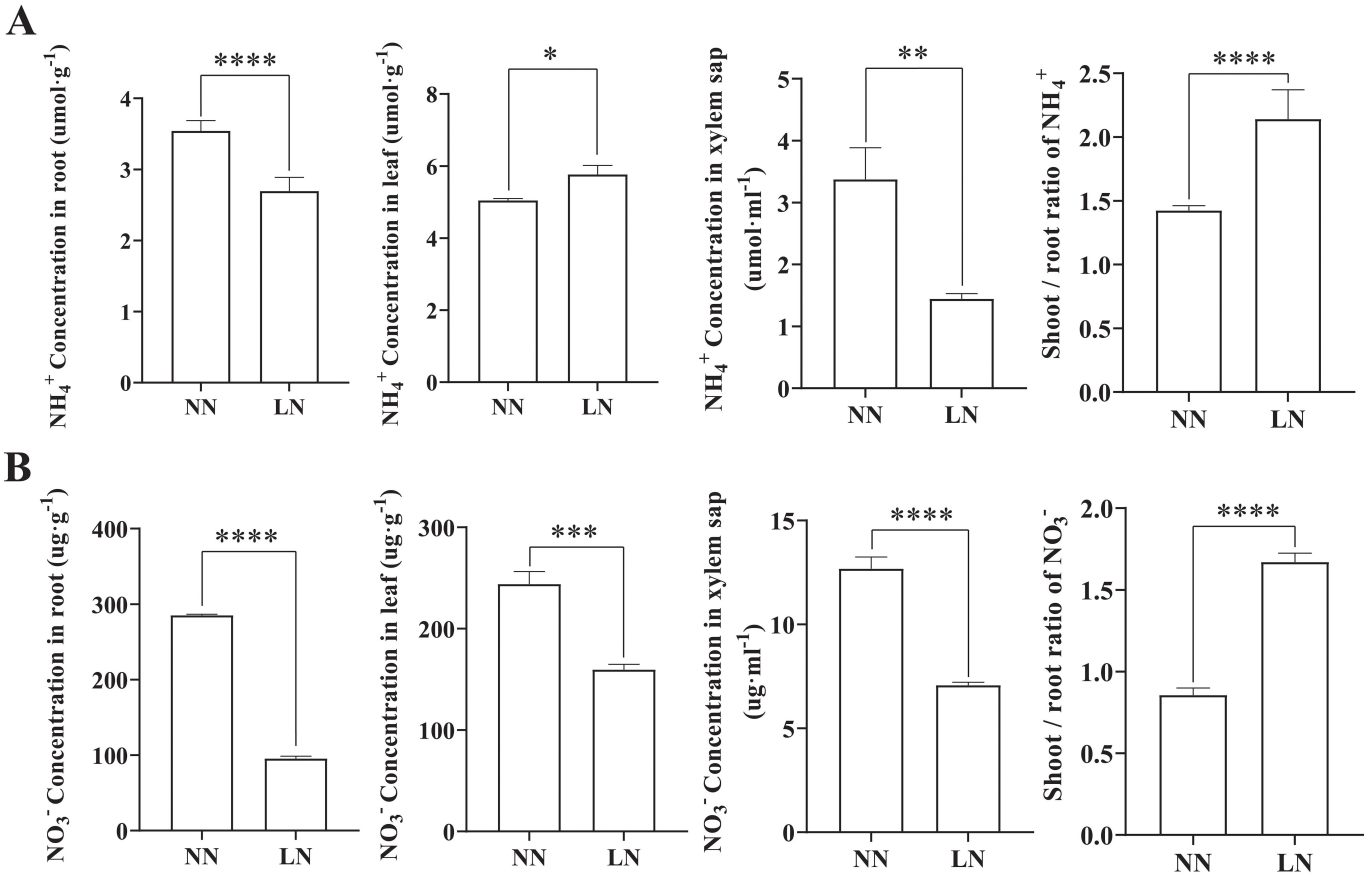
Effects of Low Nitrogen on NH_4_^+^ and NO3- Concentrations in Cassava Seedlings’ Leaves and Roots. **(A)** NH_4_^+^ concentrations in roots, leaves, and xylem sap. **(B)** NO_3_^-^ concentrations in roots, leaves, and xylem sap. Data are presented as means ± SD (n ≥ 3). * means p ≤0.05, ** means p ≤0.01, *** means p ≤0.001, **** means p ≤0.0001.

Plants absorb nitrogen primarily as NH_4_^+^ or NO_3_^-^ and transport it to above-ground tissues. To elucidate the distribution of these nitrogen forms in cassava under LN conditions, the NH_4_^+^ and NO_3_^-^ content in leaves, roots, and xylem sap were measured under both nitrogen levels (**Figure 2**). The results indicated that LN treatment significantly reduced the NH_4_^+^ and NO_3_^-^ content in cassava roots, with a more pronounced decrease in NO_3_^-^. In contrast, while LN treatment reduced NO3^-^ levels in the leaves, it induced NH_4_^+^ accumulation in the leaf tissues. Furthermore, both NH_4_^+^ and NO3^-^ levels in the xylem sap were lower in LN-treated plants. Additionally, the ratio of NH_4_^+^ and NO3^-^ content in shoots to roots (S/R) increased under LN treatment. This suggests that, in response to reduced total nitrogen availability, cassava may preferentially transport a greater proportion of NH_4_^+^ and NO_3_^-^ to leaf tissues for assimilation and utilization.

### 3.2 Physiological resistance indices

**Figure 3:**
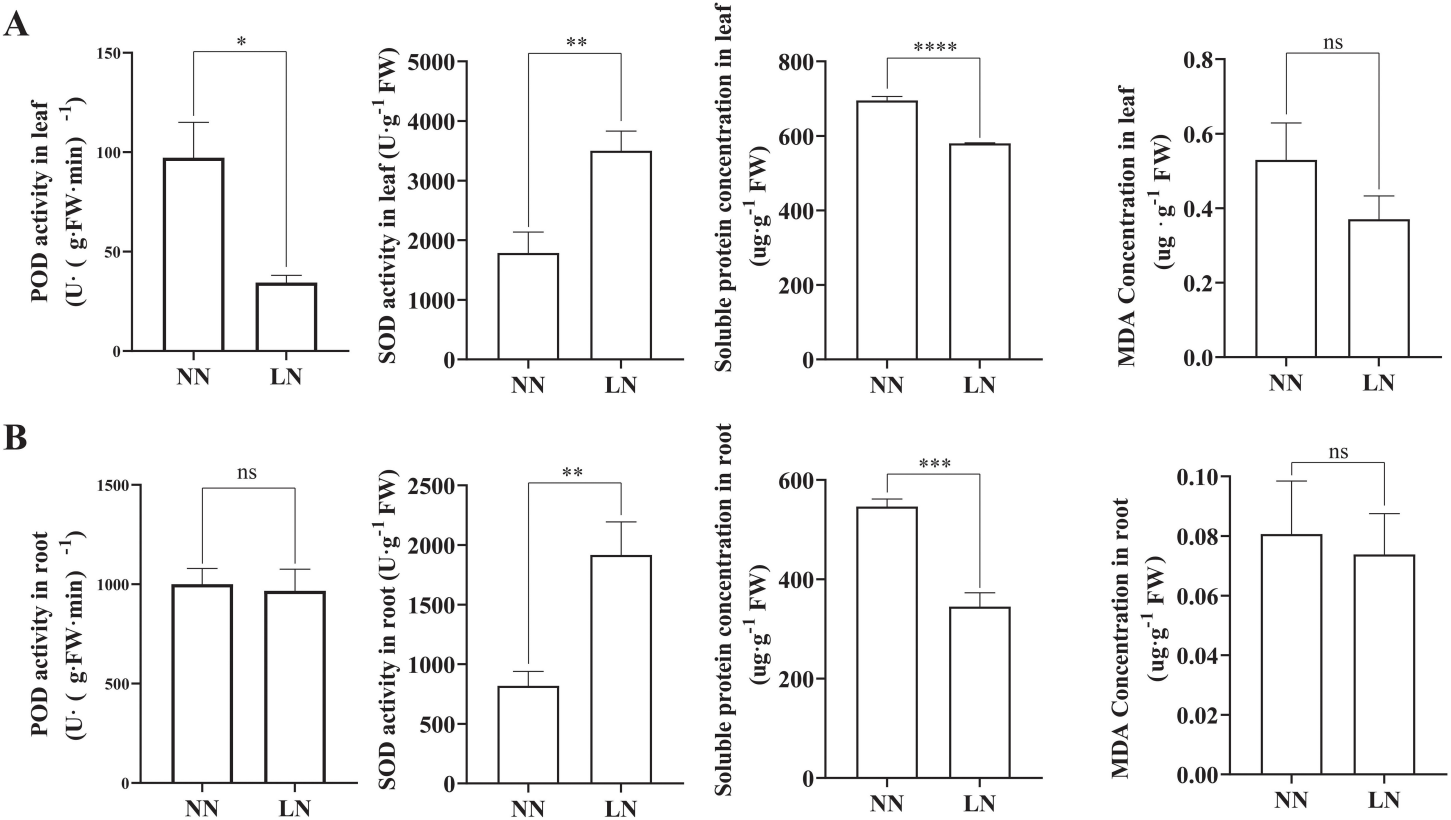
Effects of Low Nitrogen on Physiological Resistance Indices. **(A)** SOD activity, POD activity, soluble protein content, and MDA content in cassava leaves under varying nitrogen levels. **(B)** SOD activity, POD activity, soluble protein content, and MDA content in cassava roots under varying nitrogen levels. Data are presented as means ± SD (n ≥ 3). * means p ≤0.05, ** means p ≤0.01, *** means p ≤0.001, **** means p ≤0.0001 and ns means that there were no significant differences between the two groups.

LN stress significantly affected the activities of antioxidant enzymes, soluble protein levels, and MDA concentration in cassava (**Figure 3**). Compared to the NN group, the LN treatment resulted in a significant increase in SOD activity in both roots and leaves, while the soluble protein content markedly decreased. LN treatment also reduced the activity of POD and MDA content in the leaves, although these parameters remained largely unchanged in the roots.

### 3.3 Transcriptome responses of cassava leaves and roots to different nitrogen treatments

**Figure 4:**
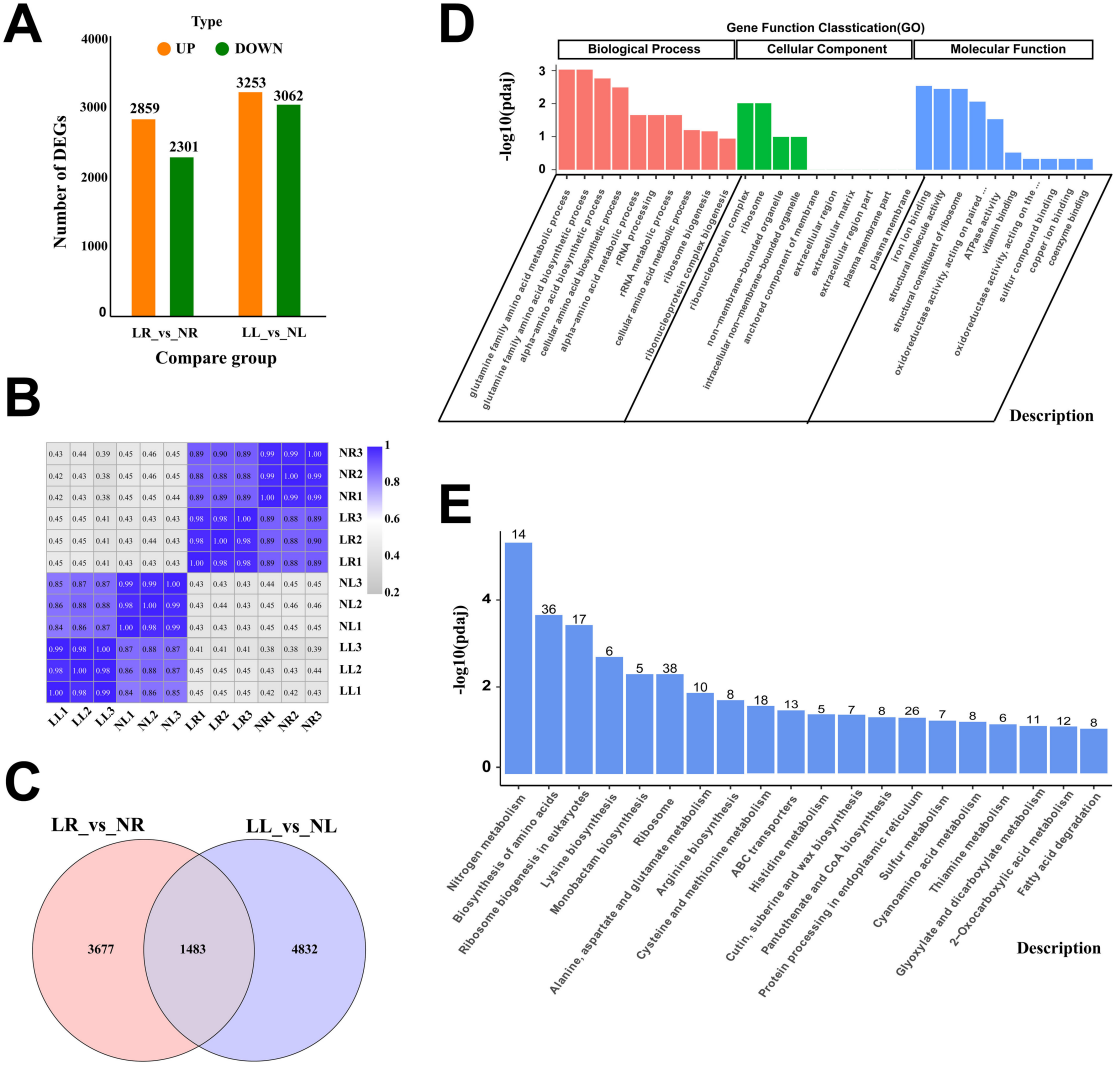
RNA-Seq Data Expression Profile of Cassava Roots and Leaves Under Low Nitrogen Stress. **(A)** Number of DEGs in different comparison groups. **(B)** Heatmap representing Pearson correlation coefficients of transcript expression between all samples. **(C)** Venn diagram of DEGs between two comparison groups. **(D)** GO enrichment analysis of DEGs common to roots and leaves. **(E)** KEGG enrichment analysis of DEGs common to roots and leaves. Data are presented as means ± SD (n ≥ 3). * means p ≤0.05, ** means p ≤0.01, *** means p ≤0.001, ****means p ≤0.0001 and ns means that there were no significant differences between the two groups.

Transcriptome sequencing was performed on four groups of samples (NL, LL, NR, and LR) subjected to two nitrogen levels. The data analysis revealed that, the clean reads for the four groups ranged from 41,821,768 to 59,891,098, with clean base counts between 6.27 and 8.98 G, whiles Q30 values ranging from 94.89% to 97.27% (**Supplementary Table S2**). Alignment with the cassava reference genome shows a total mapping range of 88.22% to 95.02%, with unique mapping rates between 85.93% and 92.54% (**Supplementary Table S3**). These results confirm the high quality of the sequencing data and the suitability of the selected reference genome. Additionally, strong correlations were observed among samples within the same treatment group (**Figure 4B**), indicating the reliability of the data for subsequent analyses.

Differentially expressed genes (DEGs) were identified based on the criteria of |log2(FoldChange)| ≥ 1 and padj ≤ 0.05. In the LR_vs_NR comparison group, 5,160 DEGs were identified, with 2,859 upregulated and 2,301 downregulated. In the LL_vs_NL comparison group, 6,315 DEGs were identified, including 3,253 upregulated and 3,062 downregulated genes. The number of upregulated DEGs in both comparison groups slightly exceeded the number of downregulated DEGs (**Figure 4A**). A comparative analysis of DEGs in roots and leaves under low nitrogen treatment revealed that, 1,483 DEGs were simultaneously altered in both tissues (**Figure 4C**), suggesting these genes may play a pivotal role in cassava’s response to low nitrogen stress. GO enrichment analysis of the co-expressed genes categorized them into three groups: biological processes (BP), cellular components (CC), and molecular functions (MF) (**Figure 4D**). The most enriched GO terms in BP were related to the glutamine family amino acid metabolic process and glutamine family amino biosynthetic process. In CC, the most enriched terms were ribonucleoprotein complex and ribosome, while in MF, the most enriched terms were iron ion binding and structural molecule activity. KEGG pathway enrichment analysis highlighted nitrogen metabolism, biosynthesis of amino acids, ribosome biogenesis in eukaryotes, and ribosome pathways as the primary metabolic pathways exhibiting co-expression (**Figure 4E**).

**Figure 5:**
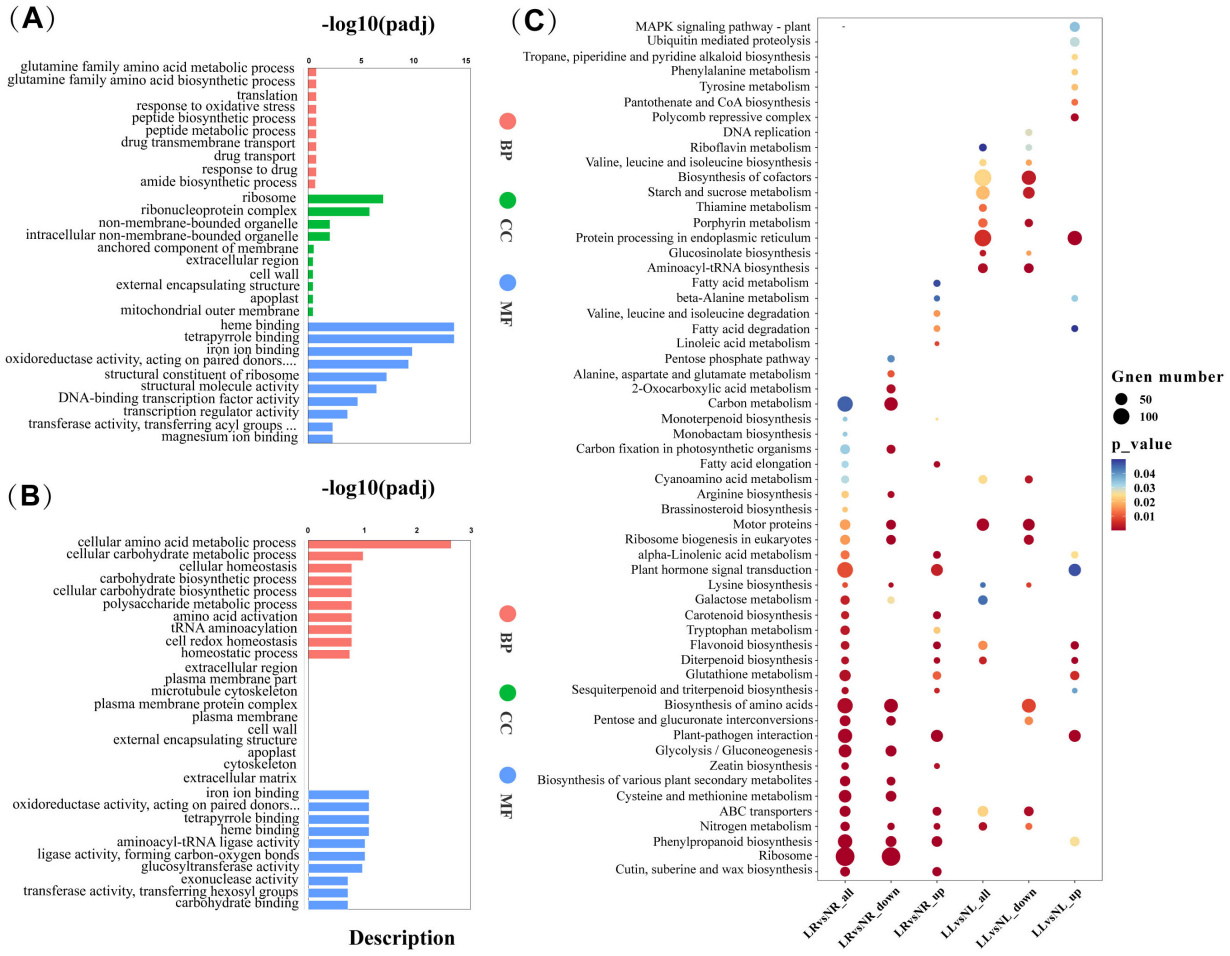
GO Functional Analysis and KEGG Pathway Analysis of DEGs During Low Nitrogen Stress. **(A)** GO terms enriched in leaves (LL_vs_NL). **(B)** GO terms enriched in roots (LR_vs_NR). **(C)** KEGG enrichment analysis of DEGs in leaves and roots. Different colors represent varying levels of enrichment significance, with red indicating more significant enrichment. The diameter of the dots represents the number of genes enriched in each pathway, with larger diameters indicating more genes.

**Figures 5A, B** illustrate the GO enrichment analysis of DEGs in the LL_vs_NL and LR_vs_NR comparison groups, respectively. In the LL_vs_NL group, DEGs are predominantly enriched in the CC and MF categories. The “ribosome” term is the most significantly enriched in the CC category, while “heme binding” and “tetrapyrrole binding” are the most significantly enriched terms in the MF category. Conversely, in the LR_vs_NR group, DEGs are mainly enriched in both BP and MF categories, with “cellular amino acid metabolic process” being the most significantly enriched term in BP, and “iron ion binding” in MF. KEGG enrichment analysis was also conducted for the upregulated and downregulated DEGs in both comparison groups. **Figure 5C** visually presents these results. In the LR_vs_NR group, downregulated DEGs are significantly enriched in pathways such as Ribosome (133), Biosynthesis of amino acids (61), Ribosome biogenesis in eukaryotes (27), Nitrogen metabolism (43), Glycolysis/Gluconeogenesis (36), and Carbon metabolism (59). Conversely, upregulated DEGs in this group are significantly enriched in pathways including Cutin, suberine, and wax biosynthesis (22), Plant-pathogen interaction (41), Carotenoid biosynthesis (15), Flavonoid biosynthesis (15), and Phenylpropanoid biosynthesis (31). In the LL_vs_NL group, downregulated DEGs are significantly enriched in pathways such as Aminoacyl-tRNA biosynthesis (27), Porphyrin metabolism (19), Biosynthesis of amino acids (62), Motor proteins (30), and Biosynthesis of cofactors (61). The upregulated DEGs in this group are significantly enriched in pathways including Protein processing in the endoplasmic reticulum (70), Flavonoid biosynthesis (17), Diterpenoid biosynthesis (11), Polycomb repressive complex (17), and Pantothenate and CoA biosynthesis (12). These results indicate that low nitrogen has a great impact on the expression of downregulated genes involved in nitrogen utilization in cassava plants, including the biosynthesis of proteins and secondary metabolites. Proteins are the material basis of cell function, and their biosynthesis requires the participation of aminoacyl-tRNA and is dominated by ribosomes composed of ribonucleoproteins (Glincy and Ingolia, 2017; Kelly and Bedwell, 2015). Ribosome and aminacyl-trna biosynthesis, eukaryotic ribosome biogenesis and other terms are the main pathways involved in downregulated DEGs in roots and leaves, combined with the fact that low nitrogen causes a significant decrease in soluble protein in cassava plants, it was speculated that low nitrogen could improve nitrogen remobilization and increase amino acid distribution under low nitrogen stress by inhibiting protein synthesis in cassava plants, thus improving cassava’s adaptability to low nitrogen. In both root and leaf, upregulated DEGs were significantly enriched in metabolic pathways related to secondary metabolite synthesis, suggesting that secondary metabolite and protein synthesis may play a synergistic role in cassava’s response to low nitrogen stress, the pathways related to Biosynthesis of amino acids, Ribosome, Flavonoid biosynthesis, Nitrogen metabolism, and Carbon metabolism are critical for cassava roots and leaves in adapting to low nitrogen stress.

### 3.4 Metabolomic responses of cassava leaves and roots to different nitrogen treatments.

To elucidate the effects of low nitrogen treatment on metabolite accumulation in cassava leaves and roots, non-targeted metabolomic analyses were conducted on four sample groups. A total of 1,579 metabolites were detected in the roots, and 1,519 in the leaves. PCA results indicated that low nitrogen treatment induced distinct changes in metabolite profiles in both cassava leaves and roots, with replicates clustering well within each treatment. PC1 accounted for 43.87% and 48.72% of the variance in the LL_vs_NL and LR_vs_NR comparisons, respectively (**Supplementary Figures S1A, D**). Applying selection criteria of VIP > 1, P value < 0.05, and FC ≥ 2 or FC ≤ 0.5, 431 differentially accumulated metabolites (DAMs) were identified in the LL_vs_NL comparison (252 upregulated, 179 downregulated), and 477 DAMs were identified in the LR_vs_NR comparison (248 upregulated, 229 downregulated) (**Supplementary Figures S1B, E**). Heatmaps of these DAMs clearly demonstrate their accumulation trends in the two comparison groups (**Supplementary Figures S1C, F**).

**Figure 6:**
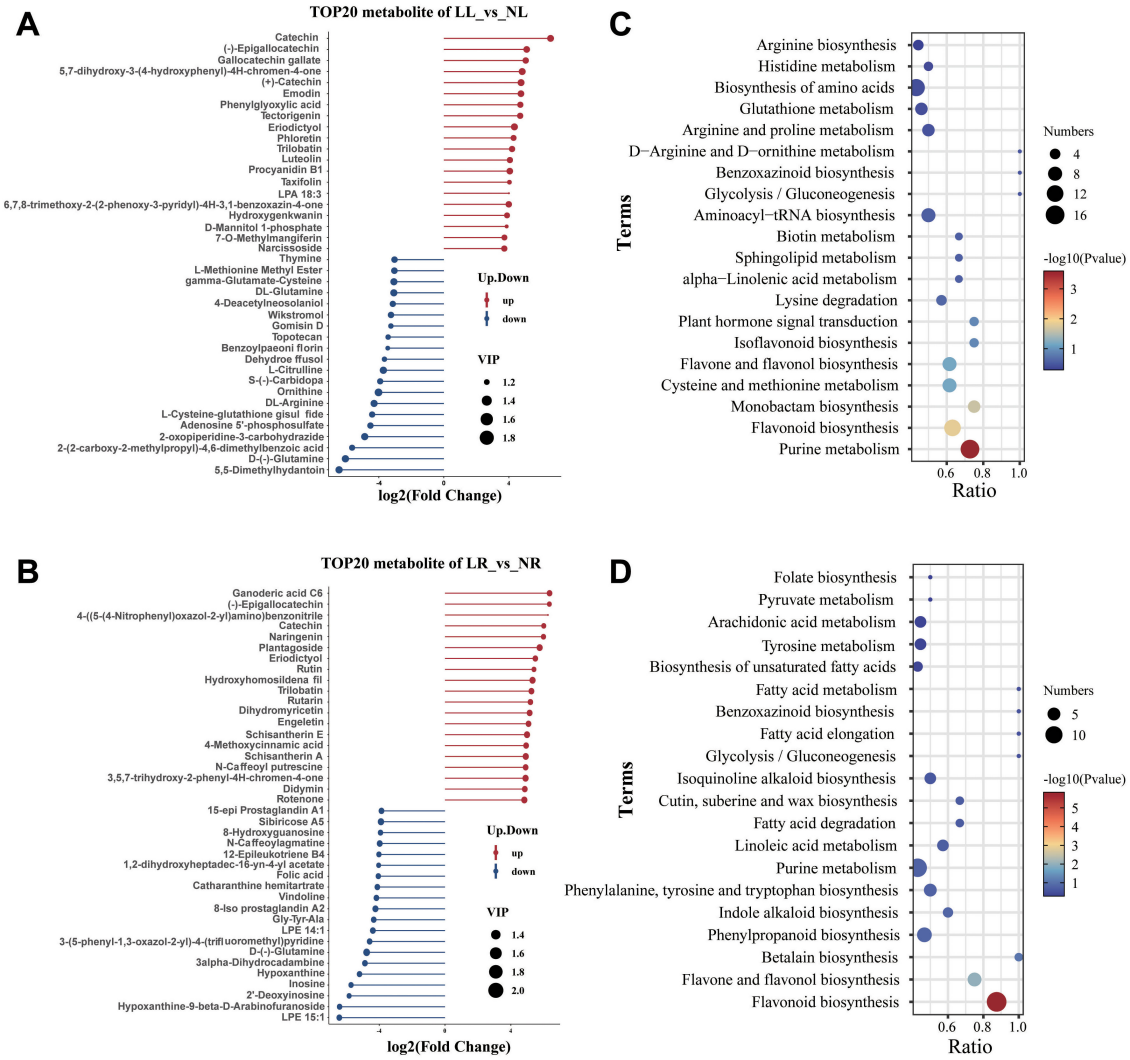
Metabolomic analysis of cassava roots and leaves under low nitrogen stress. **(A)** The top 20 metabolites with the largest upregulated or downregulated fold changes in cassava leaves under low nitrogen treatment. **(B)** Bubble chart of KEGG pathways for differentially accumulated metabolites (DAMs) in leaves (LL_vs_NL). **(C)** The top 20 metabolites with the largest upregulated or downregulated fold changes in cassava roots under low nitrogen treatment. **(D)** Bubble chart of KEGG pathways for DAMs in roots (LR_vs_NR).

A bar chart displayed the top 20 DAMs with the largest fold changes in upregulation or downregulation across the two comparison groups. In the LL_vs_NL comparison, Catechin (Com_416_neg) exhibited the greatest upregulation (Log2FC = 6.56), while 5,5-Dimethylhydantoin (Com_781_neg) showed the most significant downregulation (Log2FC = -6.44) (**Figure 6A**). In the LR_vs_NR comparison, Ganoderic acid C6 (Com_2436_neg) had the highest upregulation (Log2FC = 6.35), whereas LPE 15:1 (Com_4442_neg) demonstrated the largest downregulation (Log2FC = -6.42) (**Figure 6B**). Notably, several of the most significantly upregulated or downregulated metabolites in both comparison groups were flavonoids, including Catechin, Gallocatechin gallate, (+)-Catechin, Naringenin, Dihydromyricetin, Eriodictyol, Rutin, Luteolin, and Taxifolin, all of which showed significant increases in concentration following low nitrogen treatment. Conversely, metabolites involved in nitrogen metabolism, such as DL-Glutamine, L-Citrulline, Ornithine, D-(-)-Glutamine, and DL-Arginine, exhibited substantial decreases, particularly in the leaves. This suggests that low nitrogen treatment disrupts metabolic pathways related to nitrogen metabolism and flavonoid biosynthesis. To validate this hypothesis, KEGG enrichment analysis was performed on the DAMs. The results confirmed that DAMs in both roots and leaves were significantly enriched in the flavonoid biosynthesis and flavone and flavonol biosynthesis pathways. Additionally, the purine metabolism pathway was significantly affected by low nitrogen treatment in cassava roots, while many of the other DAMs were associated with metabolic pathways involved in amino acid synthesis (**Figures 6C, D**).

### 3.5 The impact of low nitrogen on the carbon and nitrogen metabolism pathways in cassava seedlings

**Figure 7:**
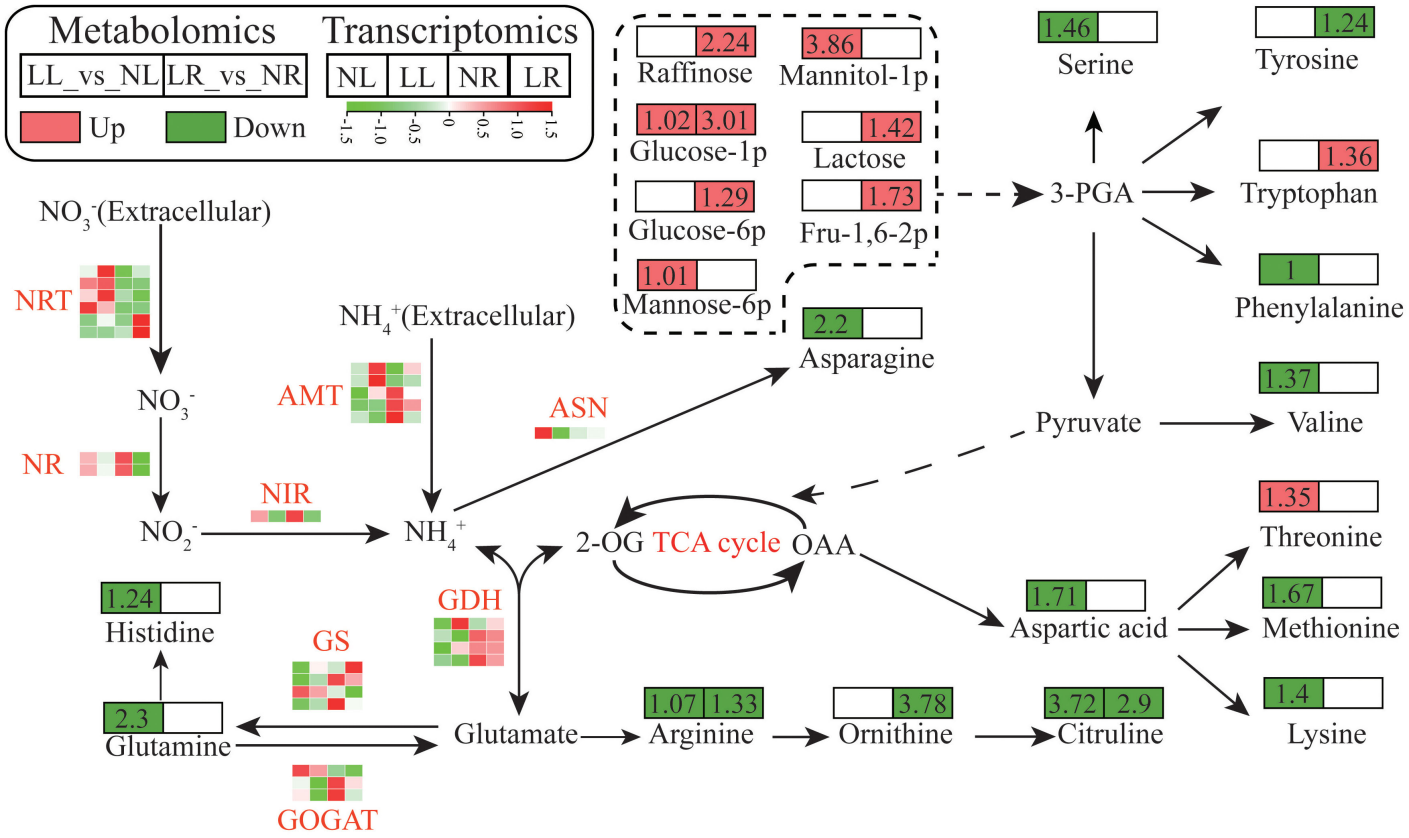
Schematic diagram of carbon and nitrogen metabolism pathways in cassava seedlings under Low Nitrogen (LN) and Normal Nitrogen (NN) Treatments. The fold change of each metabolite is indicated in the square above the metabolite, with red representing upregulation, green representing downregulation, and colorless indicating no change or undetected metabolites. Gene expression levels are depicted using a heat map, where red indicates higher expression abundance.

Under low nitrogen treatment, significant alterations were observed in genes related to nitrogen uptake and assimilation, as well as in the levels of amino acids and carbohydrates. Based on literature and KEGG pathway analysis, a schematic diagram was created to illustrate the changes in carbon and nitrogen metabolism pathways in cassava under low nitrogen stress (**Figure 7**; **Supplementary Tables S6**, **S7**). Seven DEGs encoding high-affinity nitrate transporters (NRT) and five DEGs encoding ammonium transporters (AMT) were identified in the leaves and roots. Among these, four NRTs were upregulated in the leaves, and two were significantly upregulated in the roots under low nitrogen conditions. Conversely, two AMTs were upregulated in the leaves, while three were downregulated in the roots. Additionally, genes encoding nitrate reductase (NR, 2 DEGs), nitrite reductase (NIR, 1 DEG), asparagine synthetase (ASN, 1 DEG), and glutamate synthase (GOGAT, 3 DEGs) were all significantly downregulated in both leaves and roots under low nitrogen treatment. Four DEGs encoding GS displayed varying expression patterns: three were significantly downregulated in both roots and leaves, while one was upregulated. The expression patterns of these genes were validated using qRT-PCR, which showed a strong correlation (r^2^ = 0.9412) with the transcriptome data, further confirming the reliability of the transcriptomic analysis (**Supplementary Figure S2**). Under low nitrogen stress, the content of most amino acids detected in the roots and leaves was significantly reduced, while the concentration of several key carbohydrates notably increased. Additionally, starch content in both roots and leaves was assessed, revealing that low nitrogen levels induced starch accumulation in cassava, with a higher concentration observed in the leaves. Notably, the starch content in the LL treatment group was more than double that of the NL treatment group (**Supplementary Figure S3**).

### 3.6 Transcriptomic and metabolomics analyses revealed contrasting patterns of flavonoid biosyntheses in response to low nitrogen

**Figure 8:**
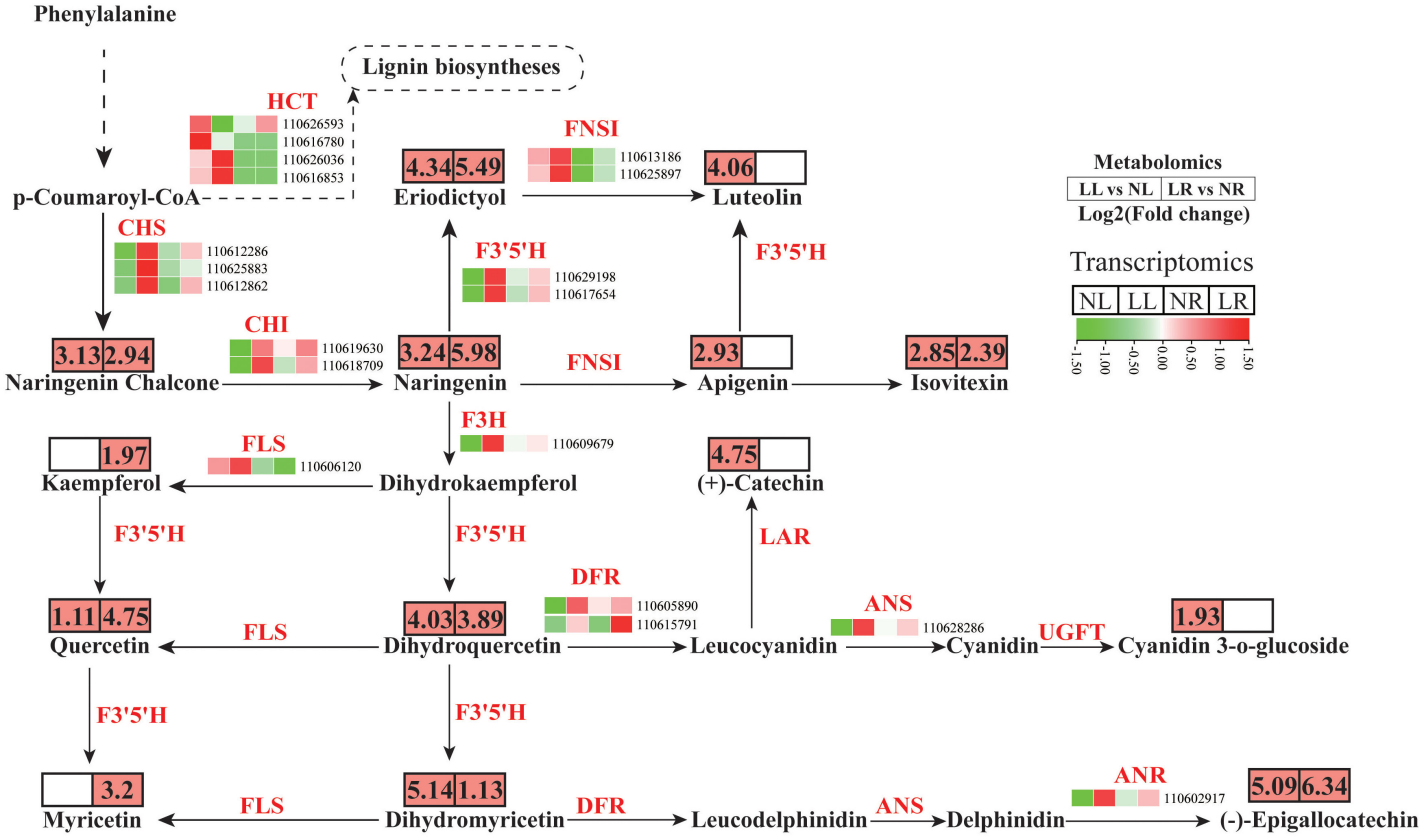
Metabolite and transcript abundances in the flavonoid biosynthesis pathway in cassava roots and leaves under low nitrogen treatment.

The phenylpropanoid biosynthesis pathway encompasses both the flavonoid and lignin biosynthesis pathways, with p-Coumaroyl-CoA acting as a common substrate for these two parallel branches. Under low nitrogen conditions, no significant changes were observed in the concentration of caffeic acid or other metabolites associated with the lignin pathway in cassava roots and leaves. The HCT gene encodes Hydroxycinnamoyl-CoA shikimate/quinate hydroxycinnamoyl transferase, a critical enzyme that introduces substrates into lignin biosynthesis (Dong and Lin, 2020). Four DEGs encoding HCT were identified in cassava roots and leaves under low nitrogen conditions, with their expression predominantly occurring in the leaves (**Figure 8**; **Supplementary Tables S6**, **S7**). Among these, two genes (110626593 and 110616780) were significantly downregulated in cassava leaves subjected to low nitrogen treatment, while the other two were upregulated. Despite the lack of significant changes in the abundance of lignin biosynthesis metabolites, it is speculated that cassava may regulate lignin content under low nitrogen conditions by modulating the expression of HCT genes.

In the flavonoid biosynthesis pathway, 15 DEGs were detected, with 14 showing coordinated changes. These include three chalcone synthase (CHS) genes, two chalcone isomerase (CHI) genes, two flavonoid 3’,5’-hydroxylase (F3’5’H) genes, one flavanone 3-hydroxylase (F3H) gene, one flavonol synthase (FLS) gene, one dihydroflavonol reductase (DFR) gene, one anthocyanin synthase (ANS) gene, and one anthocyanidin reductase (ANR) gene. These genes were significantly upregulated in both leaves and roots under low nitrogen treatment, with higher baseline expression levels and fold increases observed in the leaves compared to the roots. Additionally, another DEG encoding DFR was significantly upregulated specifically in the roots under low nitrogen conditions. Correspondingly, the concentrations of metabolites associated with these genes also significantly increased. A total of 14 DAMs linked to the flavonoid biosynthesis pathway or its downstream pathways were detected in cassava leaves and roots under low nitrogen treatment. These metabolites include naringenin chalcone, naringenin, eriodictyol, luteolin, apigenin, isovitexin, kaempferol, (+)-catechin, quercetin, dihydroquercetin, cyanidin 3-o-glucoside, (-)-epigallocatechin, dihydromyricetin, and myricetin. These metabolites were upregulated in at least one tissue following low nitrogen treatment. Overall, low nitrogen stress led to alterations in the flavonoid composition of cassava, likely due to changes in the expression levels of associated structural genes.

### 3.7 Prediction and screening of transcription factors involved in flavonoid biosynthesis

**Figure 9:**
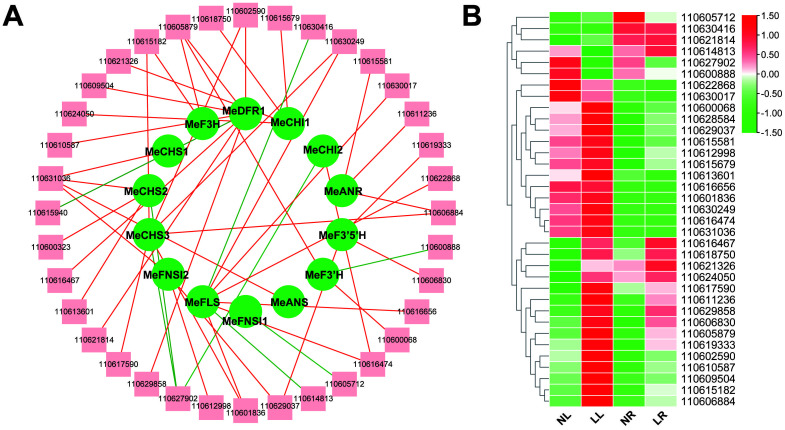
Transcription factor prediction based on RNA-seq data. **(A)** Structural genes related to flavonoid synthesis and their corresponding candidate transcription factors based on RNA-seq data. Edges colored in red and green represent positive and negative correlations, respectively, as determined by Pearson correlation coefficient (PCC) > 0.8 or PCC < −0.8. The gene names of the transcripts are listed in **Supplementary Table S5**. **(B)** Heat map of candidate transcription factors.

Transcription factors (TFs) are pivotal in regulating the transcriptional control of flavonoid biosynthesis pathways and play a pivotal role in the accumulation of flavonoids (Dong and Lin, 2020). To gain deeper insights into the mechanisms driving flavonoid synthesis, it is essential to identify the TFs involved in the expression of flavonoid-related structural genes. Using PlantRegMap (Tian et al., 2020) (http://plantregmap.gao-lab.org/), potential TFs for the 15 DEGs in the flavonoid biosynthesis pathway were predicted. Given that a single transcription factor can regulate multiple genes, duplicate predictions were eliminated, resulting in the identification of 186 TFs predicted to bind to the promoter regions of these 15 structural genes (**Supplementary Table S4**). Subsequent Pearson correlation analysis was conducted to assess the expression levels of the predicted TFs in relation to their corresponding target genes. Transcription factors with a high correlation to structural gene expression were retained, specifically those with a Pearson correlation coefficient (PCC) of |r|>0.8. This analysis ultimately predicted 37 candidate TFs likely to act on the promoters of 14 flavonoid biosynthesis structural genes (**Supplementary Table S5**). These TFs are distributed across 16 transcription factor families, with the majority belonging to the bHLH, MYB, ERF, and bZIP families. A network diagram was constructed to illustrate the regulatory relationships between transcription factors and structural genes (**Figure 9A**). This diagram highlights the significant positive or negative correlations between these transcription factors and the expression levels of their corresponding target genes. **Figure 9B** shows the expression abundance of these TFs. These findings suggest that these transcription factors are likely involved in the regulation of genes associated with flavonoid biosynthesis.

## 4 Discussion

### 4.1 Low nitrogen availability significantly inhibited cassava growth

Nitrogen (N) is a critical factor for maintaining plant growth and productivity. Previous studies have demonstrated that, root morphology is closely associated with environmental nitrogen availability, with moderate low nitrogen conditions often promoting root elongation to enhance nutrient acquisition (Wang et al., 2023b). However, in this study, low nitrogen treatment inhibited the growth of both cassava roots and aboveground parts, with a significant reduction in total root length and root volume. This suggests that cassava may possess root adaptability mechanisms that differ from those of other plants, at least in the variety examined.

### 4.2 Low nitrogen availability affected the expression of genes related to nitrogen absorption in cassava

Under low nitrogen stress, plants optimize the use of limited nitrogen by regulating nitrogen absorption assimilating related processes, there by accelerating internal nitrogen cycling (Gao et al., 2022). Nitrate is one of the primary forms of inorganic nitrogen available to plants (Wang et al., 2012). Plants absorb nitrate from the soil through high-affinity nitrate transporters (NRTs), which is then reduced to ammonium *via* the sequential actions of nitrate reductase (NR) and nitrite reductase (NIR) before entering amino acid assimilation processes (Xu et al., 2012). Under nitrogen-limited conditions, plants enhance their nitrogen acquisition capacity by regulating the expression of high-affinity nitrate transport systems (HATs) (Zhuo et al., 2010; Kiba et al., 2012; Lezhneva et al., 2014). In this study, six low-nitrogen-responsive NRTs were identified, with two showing significant upregulation in cassava roots under LN treatment. This indicates that these two NRTs may play a critical role in nitrate absorption in cassava under low nitrogen conditions (**Figure 7**). The remaining four NRTs are primarily expressed in leaves, where they may contribute to nitrate transport or function as signaling molecules. Ammonium is another form of inorganic nitrogen that plants can absorb and utilize. From the perspective of reducing equivalents and metabolic costs, it is more advantageous for plants to absorb ammonium under nitrogen deficiency, as the absorption and assimilation of ammonium nitrogen consume less energy compared to nitrate nitrogen (Andrews et al., 2013). However, excessive ammonium can be toxic to plants, necessitating precise regulation of its absorption and transport by high-affinity ammonium transporters (AMTs) located in the plasma membrane (Britto and Kronzucker, 2002). In cassava, five AMT genes were identified as being influenced by low nitrogen stress. Of these, two genes were significantly upregulated in the leaves under low nitrogen treatment. Given the accumulation of NH_4_^+^ in cassava leaves under these conditions, it is speculated that these two AMTs may be involved in the retrieval of ammonium from photorespiration escaping mitochondria and the import of ammonium in the xylem. This suggests that cassava enhances its ammonium storage capacity by optimizing the recovery and transport of ammonium under nitrogen-deficient conditions, thereby improving the utilization of limited nitrogen resources. Additionally, the other three AMTs exhibited downregulated expression in cassava roots under low nitrogen treatment, consistent with findings from a previous study on *Medicago truncatula* (Straub et al., 2014).

### 4.3 Low nitrogen availability resulted in decreased nitrogen assimilation ability of cassava

Genes involved in nitrogen assimilation are regulated by external nitrogen levels, directly influencing the plant’s metabolic processes. The glutamine synthetase (GS)/glutamine-2-oxoglutarate aminotransferase (GOGAT) cycle plays a central role in assimilating absorbed NH_4_^+^ into glutamate and glutamine, which are essential precursors for the synthesis of other amino acids (Xu et al., 2012). Cytosolic asparagine synthetase (AS) further catalyzes the production of asparagine and glutamate from glutamine and aspartate (Sichul et al., 2020). NADH-dependent glutamate dehydrogenase (GDH) facilitates the reversible deamination of glutamate (Glu) to yield ammonium and 2-oxoglutarate (2-OG), essential for maintaining carbon and nitrogen metabolism balance in plants (Gao et al., 2022). In cassava, the expression of 15 genes related to nitrogen assimilation—including NR (2), NIR (1), ASN (1), GDH (4), GS (4), and GOGAT (3)—is influenced by nitrogen availability. Most of these genes were significantly downregulated in at least one tissue under low nitrogen treatment, indicating that inadequate nitrogen supply reduces substrate availability, thereby decreasing cassava’s nitrogen assimilation capacity. This reduction consequently impacts the synthesis and accumulation of various downstream amino acids. Carbon and nitrogen metabolism are interconnected pathways essential for plant growth. Carbon metabolism provides the carbon sources and energy needed for nitrogen metabolism, while nitrogen metabolism supplies enzymes and photosynthetic pigments essential for carbon metabolism (Alisdair et al., 2010). When nitrogen metabolism pathways are obstructed, carbon sources cannot be effectively utilized, leading to the accumulation of carbon metabolism-associated metabolites, such as glucose-1-phosphate and starch, in cassava roots and leaves under low nitrogen conditions. The accumulation of starch in cassava leaves significantly inhibits photosynthesis, resulting in reduced chlorophyll content compared to normal nitrogen conditions. This inhibition leads to increased electron transfer to molecular oxygen (O_2_) *via* the Mehler reaction, generating a substantial amount of reactive oxygen species (O_2_^-^) (Podgórska et al., 2020). SOD and POD are key antioxidants that mitigate oxidative stress by removing O_2_^-^ and H_2_O_2_, respectively (Takahashi and Badger, 2011). Our research shows that low nitrogen conditions enhance SOD activity in cassava roots and leaves compared to normal nitrogen levels. However, while POD activity remained unchanged in roots, it decreased in leaves, suggesting that other enzymes, such as catalase (CAT) and ascorbic acid (ASA), may be involved in H_2_O_2_ decomposition under these conditions.

### 4.4 Low nitrogen availability significantly promoted the accumulation of flavonoids in cassava

The accumulation of secondary metabolites is a key factor for plants in adapting to and resisting low nitrogen stress. Under such conditions, the levels of certain compounds typically increase, as previous studies have shown that genes, proteins, and metabolites involved in phenylpropanoid biosynthesis are vital for plant tolerance to low nitrogen conditions (Liang and He, 2018). The aromatic amino acid phenylalanine (Phe) serves as a key substrate for the biosynthesis of lignin and flavonoids. Research has demonstrated that lignin metabolism plays a role in regulating root structure and physiological characteristics under varying nitrogen conditions (Xin et al., 2019), and flavonoid production may be linked to the scavenging of ROS during stress (Santelia et al., 2008). Low nitrogen levels have been shown to promote flavonoid accumulation in *snow chrysanthemum*, thereby enhancing the plant’s antioxidant activity (Li et al., 2023). Similar findings have been observed in grapes, rapeseed, and tomatoes, where low nitrogen conditions boost the expression of genes in the flavonoid biosynthetic pathway, leading to increased metabolite accumulation (Koeslin-Findeklee et al., 2015; Soubeyrand et al., 2014; Løvdal et al., 2010). The results of this study reveal that under low nitrogen stress, 15 DEGs and 14 DAMs are associated with the flavonoid biosynthetic pathway. Most of these genes were significantly upregulated in cassava leaves subjected to low nitrogen treatment, and flavonoid metabolites in cassava roots.The leaves also showed significant accumulation following this treatment. CHS and CHI are key enzymes in the flavonoid biosynthetic pathway, initiating substrate flow into flavonoid metabolism (Dong and Lin, 2021). Transcriptome analysis identified three low-nitrogen-induced *CHSs* and two *CHIs* in cassava, all of which exhibited significant upregulation in the leaves under low nitrogen conditions. Correspondingly, the levels of naringenin chalcone and naringenin also increased significantly. This suggests that CHS and CHI play critical roles in cassava’s flavonoid biosynthesis under low nitrogen conditions. The distribution of carbon flux under low nitrogen stress may be a key factor in flavonoid accumulation. Low nitrogen levels can create a bottleneck in plant carbon metabolism, causing carbon produced through photosynthesis to be redirected into the phenylpropanoid metabolism *via* glycolysis or the pentose phosphate pathway. This shift enhances the metabolic flux of the downstream flavonoid pathway, leading to increased flavonoid accumulation (Xin et al., 2019). Furthermore, some transcription factors (TFs) are involved in regulating flavonoid biosynthesis under low nitrogen conditions. A recent study has shown that *MdbHLH130* can directly regulate key genes related to lignin and flavonoid biosynthesis, modulating the allocation of substrates between these two parallel pathways. Inhibiting *MdbHLH130* can enhance flavonoid biosynthesis and improve nitrogen absorption efficiency (Wang et al., 2023). In this study, 37 potential TFs were identified that may regulate the expression of genes involved in flavonoid synthesis, displaying either positive or negative correlations with their target genes. These TFs may positively or negatively influence flavonoid biosynthesis. However, further experiments, such as luciferase reporter assays or yeast one-hybrid assays, are necessary to validate the functions of these candidate TFs.

## Data Availability

The data presented in the study are deposited in the Genome Sequence Archive in National Genomics Data Center, accession number CRA021513.
