# Supplementary material for: Integrated transcriptomics and metabolomics analyses provide new insights into cassava in response to nitrogen deficiency
Source: Front Plant Sci. 2025 Jan 14;15:1488281. doi: 10.3389/fpls.2024.1488281 (PMC11772423; doi:10.3389/fpls.2024.1488281)
Supplement: Supplementary file 1 [file DataSheet1.docx]

Supplementary Material

# Supplementary Figures and Tables

## Supplementary Figures


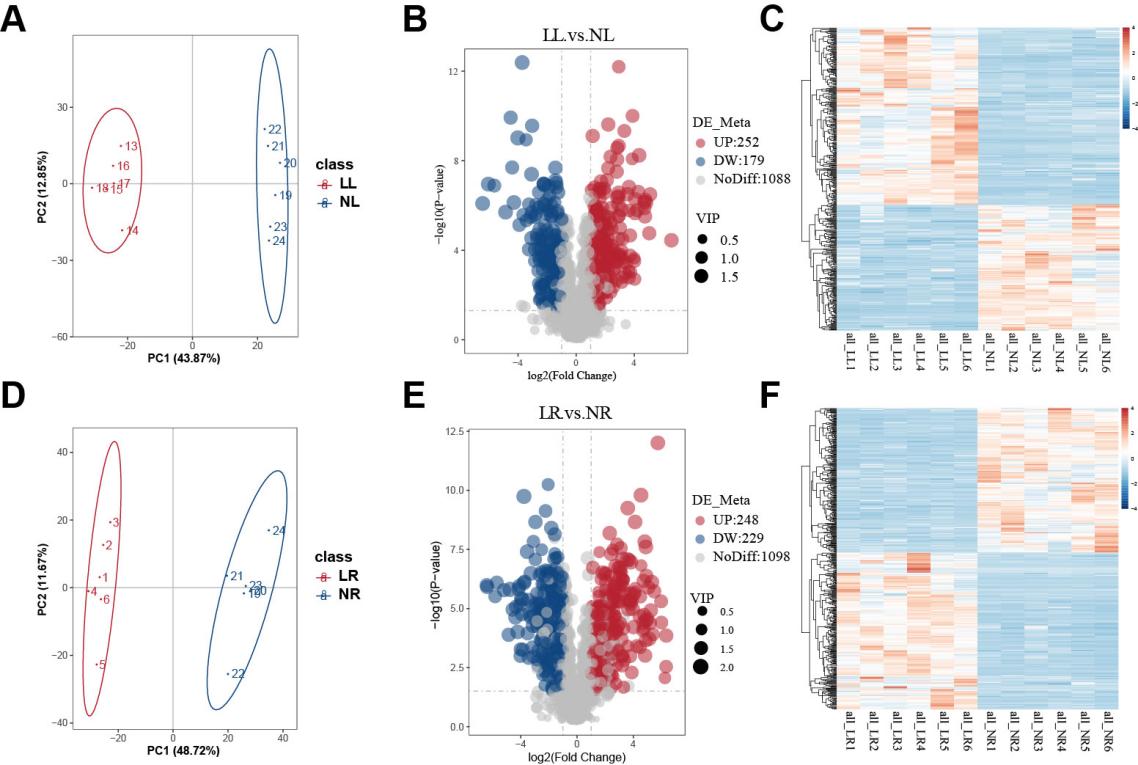


**Supplementary Figure 1. Overview of the Cassava Roots and Leaves Metabolome in Response to Low Nitrogen.** (A) PCA analysis of the LL_vs_NL comparison group. (B) Volcano plot of the LL_vs_NL comparison group. (C) Clustering heat map of total differential metabolites in the LL_vs_NL comparison group. (D) PCA of the LR_vs_NR comparison group. (E) Volcano plot of the LR_vs_NR comparison group. (F) Clustering heat map of total differential metabolites in the LR_vs_NR comparison group.


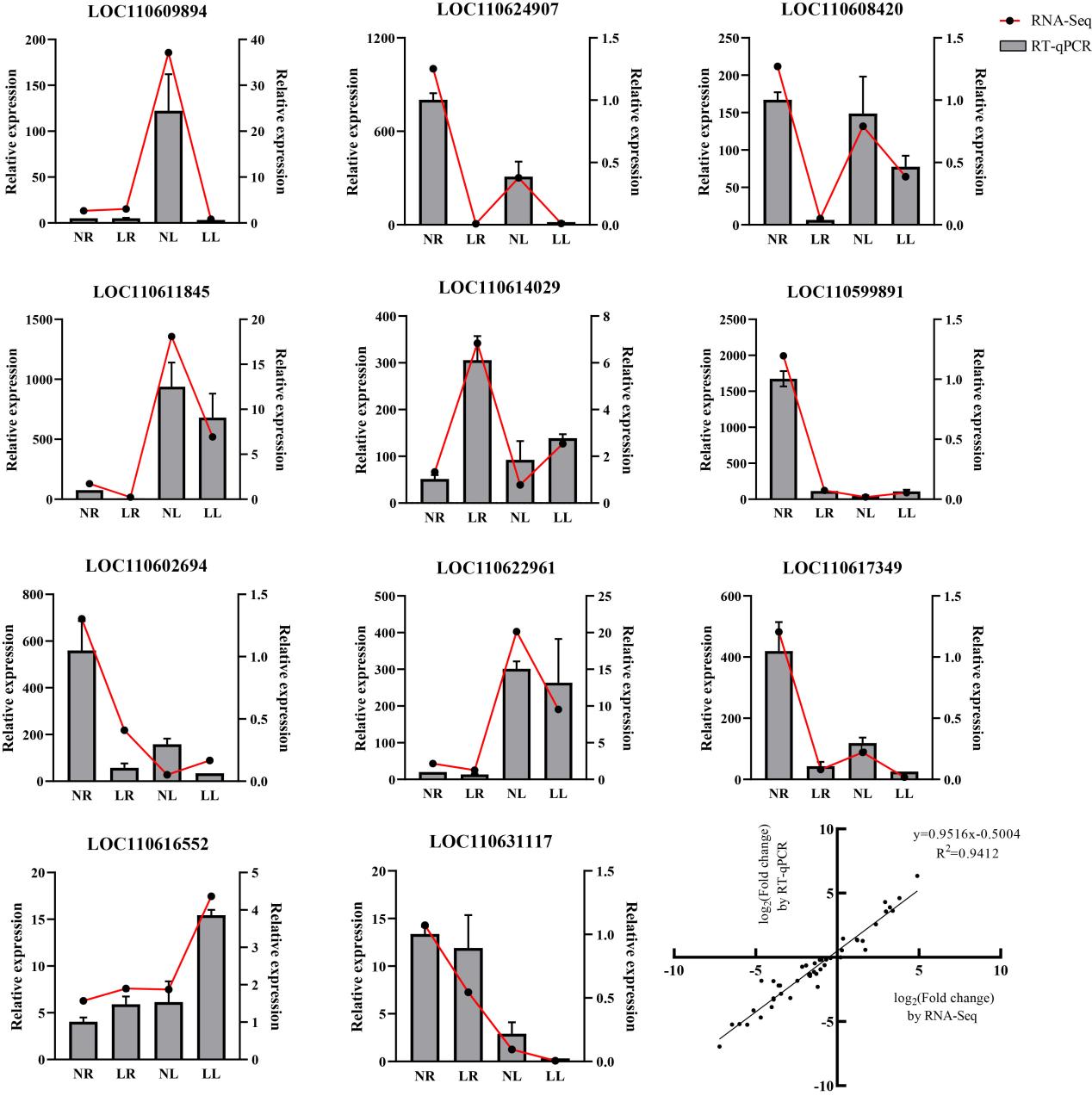


**Supplementary Figure 2. qRT-PCR Verification of the Expression of 12 Selected DEGs.** The red line in each graph represents the FPKM value obtained from RNA-seq data. Error bars indicate the standard deviation of three independent replicates. A scatter plot is provided to show the correlation between the fold change in RNA-seq and qRT-PCR for each gene.


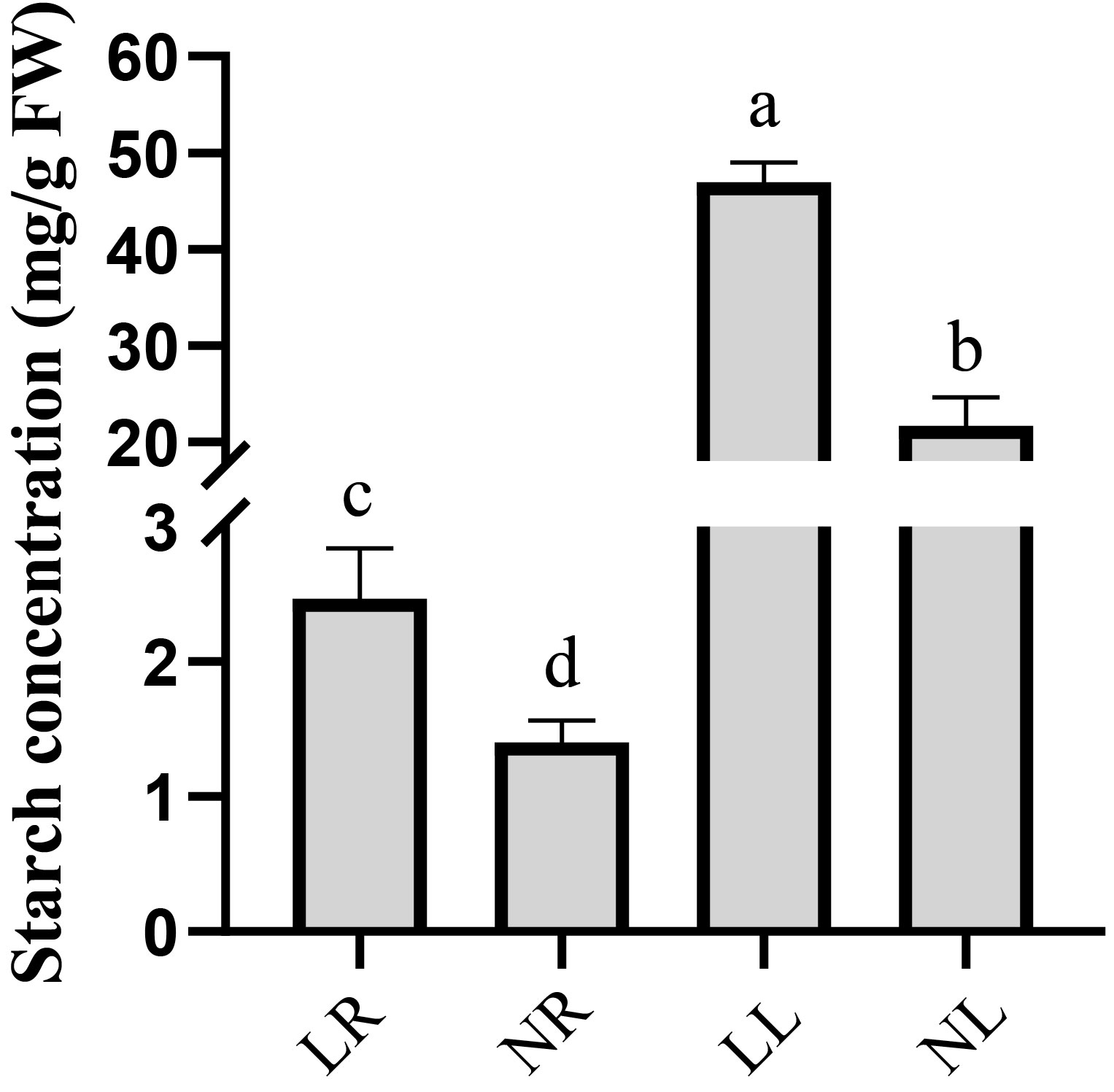


**Supplementary Figure 3. Starch Content in Cassava Leaves and Roots Under Low Nitrogen (LN) and Normal Nitrogen (NN) Treatments.** Error bars indicate the standard deviation of three independent replicates. Different letters denote significant differences according to ANOVA and Duncan's test (P < 0.05).

## Supplementary Tables

| **Supplementary Table S1. Primers used in this study** | | | | |
| --- | --- | --- | --- | --- |
| **Gene name** | **locus ID** | **Forward primers** | **Reverse primers** |  |
| MeASN1 | 110609894 | ATGGGATGCTGCGTGGTC | TGCAGCAGCTACCTGTGG |  |
| MeGDH2;1 | 110631117 | GAGGCTGCAACTGGTCGT | CCTTGATGCCCAGGACCC |  |
| MeGDH2;2 | 110616552 | AGGGATTGGTTGCAGCCC | CCATGTCTGGCGCTGGAA |  |
| MeGLT1;1 | 110617349 | GCCACGACCACCCGTTAA | TTGATCAGCAGCGGCCAA |  |
| MeGLU1 | 110622961 | ATGGCTGCAGCAATGGGT | ACTGGCAACACCGACAGG |  |
| MeGLN1;1 | 110602694 | AAGTTGGGGCTCAGGCAC | ATGCTCCACGGTTGGCAA |  |
| MeGLN1;2 | 110599891 | AGGCAATGAACGCAGGCT | GAATGGATGCTCCGCGGT |  |
| MeGLN1;3 | 110614029 | AAGCTGGGTCTGCGTCAC | TCCCCTGTTTGCAACCCC |  |
| MeGLN2 | 110611845 | TGGGTTGGCCTGTTGGAG | CCCCGTTGGTGCCACTAA |  |
| MeNIR1 | 110624907 | GGCATGTACAGGCAGCCA | AGTGCATTCTCACCGGCC |  |
| MeNR1 | 110608420 | GGTGCTTCTGGTCCCTGG | CCCGGAACCAGCAGTTGT |  |
| MeUbq9 | 110600071 | GCCTCCCAAGGTAGCTTTCA | GGTTAATGCAGGGCTCCACT |  |

| **Supplementary Table S2. A comparison of the sequencing quality of 12 libraries** | | | | | | | | |
| --- | --- | --- | --- | --- | --- | --- | --- | --- |
| **sample** | **raw_reads** | **raw_bases** | **clean_reads** | **clean_bases** | **error_rate** | **Q20** | **Q30** | **GC_pct** |
| LL1 | 49669390 | 7.45G | 48683394 | 7.3G | 0.01 | 99.01 | 97 | 43.85 |
| LL2 | 59891098 | 8.98G | 58544030 | 8.78G | 0.01 | 98.3 | 95.07 | 44.1 |
| LL3 | 41821768 | 6.27G | 40865038 | 6.13G | 0.01 | 98.47 | 95.58 | 44.7 |
| NL1 | 46814832 | 7.02G | 45582756 | 6.84G | 0.01 | 99.06 | 97.12 | 44.24 |
| NL2 | 47290132 | 7.09G | 46116886 | 6.92G | 0.01 | 99.04 | 97.08 | 44.26 |
| NL3 | 42269150 | 6.34G | 40522936 | 6.08G | 0.01 | 98.15 | 94.89 | 43.81 |
| LR1 | 49047648 | 7.36G | 48213778 | 7.23G | 0.01 | 99.02 | 97.03 | 43.48 |
| LR2 | 47325870 | 7.1G | 46615030 | 6.99G | 0.01 | 99.13 | 97.27 | 43.45 |
| LR3 | 47044046 | 7.06G | 46207766 | 6.93G | 0.01 | 99.1 | 97.19 | 43.49 |
| NR1 | 47156068 | 7.07G | 46062590 | 6.91G | 0.01 | 98.98 | 96.89 | 43.89 |
| NR2 | 57599132 | 8.64G | 56602480 | 8.49G | 0.01 | 98.96 | 96.78 | 43.6 |
| NR3 | 49830262 | 7.47G | 48843718 | 7.33G | 0.01 | 98.94 | 96.7 | 43.74 |

| **Supplementary Table S3. An overview of the genome mapping results.** | | | | | | | |
| --- | --- | --- | --- | --- | --- | --- | --- |
| **sample** | **total_reads** | **total_map** | **unique_map** | **multi_map** | **splice_map** | **unsplice_map** | **proper_map** |
| LL1 | 48683394 | 46259671(95.02%) | 45051149(92.54%) | 1208522(2.48%) | 16074353(33.02%) | 28976796(59.52%) | 43568234(89.49%) |
| LL2 | 58544030 | 55214441(94.31%) | 53780117(91.86%) | 1434324(2.45%) | 19928328(34.04%) | 33851789(57.82%) | 51647348(88.22%) |
| LL3 | 40865038 | 38295741(93.71%) | 36987832(90.51%) | 1307909(3.2%) | 12684055(31.04%) | 24303777(59.47%) | 35684058(87.32%) |
| NL1 | 45582756 | 43036081(94.41%) | 41857065(91.83%) | 1179016(2.59%) | 16585007(36.38%) | 25272058(55.44%) | 39759528(87.22%) |
| NL2 | 46116886 | 43644910(94.64%) | 42374549(91.89%) | 1270361(2.75%) | 16573186(35.94%) | 25801363(55.95%) | 40378432(87.56%) |
| NL3 | 40522936 | 38049576(93.9%) | 36908775(91.08%) | 1140801(2.82%) | 14769461(36.45%) | 22139314(54.63%) | 35069412(86.54%) |
| LR1 | 48213778 | 44581369(92.47%) | 43386515(89.99%) | 1194854(2.48%) | 17587804(36.48%) | 25798711(53.51%) | 41388854(85.84%) |
| LR2 | 46615030 | 43262609(92.81%) | 42126856(90.37%) | 1135753(2.44%) | 17136874(36.76%) | 24989982(53.61%) | 40120524(86.07%) |
| LR3 | 46207766 | 43018599(93.1%) | 41847831(90.56%) | 1170768(2.53%) | 17049326(36.9%) | 24798505(53.67%) | 40108046(86.8%) |
| NR1 | 46062590 | 40637881(88.22%) | 39579705(85.93%) | 1058176(2.3%) | 16419682(35.65%) | 23160023(50.28%) | 35900880(77.94%) |
| NR2 | 56602480 | 51881078(91.66%) | 50534784(89.28%) | 1346294(2.38%) | 20995733(37.09%) | 29539051(52.19%) | 47660494(84.2%) |
| NR3 | 48843718 | 44101020(90.29%) | 42965103(87.96%) | 1135917(2.33%) | 17928735(36.71%) | 25036368(51.26%) | 40417510(82.75%) |

| **Supplementary Table S4. TFs were predicted in structural genes related to flavonoid synthesis** | | | |
| --- | --- | --- | --- |
| **GeneID** | **TF ID** | **Family** | **Matched sequence** |
| LOC110602917(MeANR) | Manes.13G001800 | AP2 | TAAAAAAAGAAAAAAACAAA |
|  | Manes.13G043500 | B3 | ctgcatgca |
|  | Manes.17G047000 | B3 | ctgcatgc |
|  | Manes.18G029600 | BES1 | gtgcacgtgcaagggtcacg |
|  | Manes.18G029600 | BES1 | TTGCACGTGCACCCACCAAT |
|  | Manes.03G091500 | BES1 | TGCACGTGCAC |
|  | Manes.03G091500 | BES1 | tgcacgtgcaa |
|  | Manes.01G193400 | bHLH | gtgggtgcacgtgcaagggtc |
|  | Manes.06G080300 | bHLH | CACGTGACCCTTGC |
|  | Manes.18G050100 | bHLH | GAAGGTAAGCACGTGACCCTT |
|  | Manes.06G079000 | bHLH | CACGTGACCCTTGCA |
|  | Manes.13G043000 | bHLH | caagggtcacgtgc |
|  | Manes.17G016000 | bHLH | gcacgtgc |
|  | Manes.17G016000 | bHLH | GCACGTGC |
|  | Manes.05G022000 | bHLH | gcacgtgc |
|  | Manes.05G022000 | bHLH | GCACGTGC |
|  | Manes.01G199900 | bHLH | gcacgtgc |
|  | Manes.01G199900 | bHLH | GCACGTGC |
|  | Manes.18G050100 | bHLH | TGACCCTTGCACGTGCACCCA |
|  | Manes.13G043000 | bHLH | gtgggtgcacgtgc |
|  | Manes.15G039300 | bHLH | gtcacgtgct |
|  | Manes.15G039300 | bHLH | AGCACGTGAC |
|  | Manes.01G193400 | bHLH | caagggtcacgtgcttacctt |
|  | Manes.14G112400 | bHLH | tgcacgtgca |
|  | Manes.14G112400 | bHLH | TGCACGTGCA |
|  | Manes.18G050100 | bHLH | tgcaagggtcacgtgcttacc |
|  | Manes.01G193400 | bHLH | ACCCTTGCACGTGCACCCACC |
|  | Manes.06G079000 | bHLH | CACGTGCACCCACCA |
|  | Manes.01G193400 | bHLH | AGGTAAGCACGTGACCCTTGC |
|  | Manes.06G079000 | bHLH | cacgtgcttaccttc |
|  | Manes.06G012800 | CAMTA | gaaccgcgt |
|  | Manes.12G134300 | CAMTA | ccgcgtgca |
|  | Manes.03G174900 | CPP | acaaattttaagc |
|  | Manes.11G131600 | Dof | ttgtttgtttttttctttttt |
|  | Manes.12G095300 | Dof | tttgtttgtttttttcttttt |
|  | Manes.08G018300 | HD-ZIP | ttgaattattggt |
|  | Manes.05G041900 | MIKC_MADS | tgtttgtttttttcttttttt |
|  | Manes.05G041900 | MIKC_MADS | tttgtttgtttttttcttttt |
|  | Manes.05G041900 | MIKC_MADS | tgtttgtttgtttttttcttt |
|  | Manes.05G041900 | MIKC_MADS | tttgtttgtttgtttttttct |
|  | Manes.02G120800 | MYB | aggccgttacg |
|  | Manes.07G099800 | MYB | ttatgttaccgttag |
|  | Manes.11G127600 | MYB | tgttaccgtta |
|  | Manes.15G149100 | MYB | tcagaggccgttacgca |
|  | Manes.S051400 | MYB | aatagaaaccgaatc |
|  | Manes.12G108500 | MYB | TAACGGTAACA |
|  | Manes.08G106900 | MYB | TGCACCCACCAATAA |
|  | Manes.03G189400 | SBP | tcgtacgtcc |
|  | Manes.05G184900 | TALE | CACGTGACCCTTGCACGTGC |
| LOC110605890(MeDFR1) | Manes.10G046700 | ARF | TTTTTGTTTTAAGTCGGAAAA |
|  | Manes.02G197900 | bHLH | tgcaagttgga |
|  | Manes.08G047200 | bHLH | TCCAACTTGCA |
|  | Manes.14G013300 | bHLH | TATCCAACTTGCAT |
|  | Manes.02G178100 | bZIP | GTGACAGCTCAC |
|  | Manes.18G014400 | E2F/DP | attgtgcagcccaaa |
|  | Manes.18G040400 | ERF | CACCCATCACCGCCA |
|  | Manes.13G120400 | ERF | CACCCATCACCGCCA |
|  | Manes.07G135300 | ERF | gacagatgggatggcggtgat |
|  | Manes.06G156400 | ERF | CCATCACCGCCATCCCATCTG |
|  | Manes.18G019100 | ERF | CACCCATCACCGCCA |
|  | Manes.13G143500 | ERF | cagatgggatggcggtgatgg |
|  | Manes.11G013700 | ERF | cagatgggatggcggtgatgg |
|  | Manes.08G048500 | ERF | CCATCACCGCC |
|  | Manes.15G066800 | ERF | TTCCACCCATCACCGCCATCC |
|  | Manes.16G034200 | ERF | atggcggtgatgggtgg |
|  | Manes.01G085200 | ERF | CCATCACCGCCATCC |
|  | Manes.02G042400 | ERF | CACCCATCACCGCCATCCCAT |
|  | Manes.04G094600 | ERF | ATCACCGCCATCCCATCTGTC |
|  | Manes.18G075600 | ERF | ATGTTCCACCCATCACCGCC |
|  | Manes.13G143500 | ERF | agacagatgggatggcggtga |
|  | Manes.04G094600 | ERF | CCCATCACCGCCATCCCATCT |
|  | Manes.06G131200 | ERF | gggatggcggtgatg |
|  | Manes.03G011000 | ERF | atgggatggcggtgatggg |
|  | Manes.06G156400 | ERF | TCACCGCCATCCCATCTGTCT |
|  | Manes.15G014600 | ERF | CCATCACCGCCATCC |
|  | Manes.03G011000 | ERF | cagatgggatggcggtgat |
|  | Manes.09G146100 | ERF | tgggatggcggtgat |
|  | Manes.08G099800 | ERF | ggatggcggtgatgg |
|  | Manes.03G044100 | ERF | gatggcggtgatgg |
|  | Manes.01G085200 | ERF | CACCCATCACCGCCA |
|  | Manes.15G039700 | ERF | CACCCATCACCGCCATCCCATCTGTCTT |
|  | Manes.18G040400 | ERF | CCATCACCGCCATCC |
|  | Manes.02G208000 | ERF | CACCGCCATCCCATCTGTCT |
|  | Manes.02G042400 | ERF | CCATCACCGCCATCCCATCTG |
|  | Manes.18G019100 | ERF | CCATCACCGCCATCC |
|  | Manes.15G066800 | ERF | ATGTTCCACCCATCACCGCCA |
|  | Manes.01G245200 | ERF | tgggatggcggtgat |
|  | Manes.05G052000 | ERF | ggatggcggtgatgg |
|  | Manes.03G154500 | GATA | gggatggcggtgatg |
|  | Manes.03G154500 | GATA | atggcggtgatgggt |
|  | Manes.16G116200 | HSF | CCAGAGGCTTC |
|  | Manes.12G000100 | LBD | CCATCACCGCCATCCCATCTG |
|  | Manes.06G108700 | LBD | ATCACCGCCATCCCATCTGTC |
|  | Manes.01G263800 | MIKC_MADS | TTTCTGAAAAAAGAAAAGT |
|  | Manes.07G011600 | MIKC_MADS | ttttcttttttcagaaatt |
|  | Manes.02G046100 | MYB | GTTCCACCCATCACC |
|  | Manes.13G107900 | MYB | cggtgatgggtgg |
|  | Manes.13G107900 | MYB | ggagggcaggtga |
|  | Manes.08G106900 | MYB | ATCACCTACCAGTTC |
|  | Manes.18G079600 | NAC | TACGTATCCAACTTGCATTACT |
| LOC110606120(MeFLS) | Manes.06G079000 | bHLH | CACGTGACTGTCATG |
|  | Manes.18G050100 | bHLH | catgacagtcacgtggcaatt |
|  | Manes.09G181800 | bHLH | TGCCACGTGA |
|  | Manes.06G080300 | bHLH | CACGTGACTGTCAT |
|  | Manes.13G043000 | bHLH | tgacagtcacgtgg |
|  | Manes.15G039300 | bHLH | gtcacgtggc |
|  | Manes.15G039300 | bHLH | GCCACGTGAC |
|  | Manes.01G269700 | bHLH | CCACGTGA |
|  | Manes.01G193400 | bHLH | AAATTGCCACGTGACTGTCAT |
|  | Manes.12G104100 | bHLH | GCCACGTGA |
|  | Manes.18G048100 | bZIP | gacagtcacgtggca |
|  | Manes.15G191900 | bZIP | gacagtcacgtggca |
|  | Manes.18G037900 | bZIP | CAAATTGCCACGTGACTG |
|  | Manes.09G056100 | bZIP | tgacagtcacgtggcaat |
|  | Manes.01G249900 | bZIP | TGCCACGTGACTGTC |
|  | Manes.13G136900 | bZIP | tcacgtggca |
|  | Manes.08G134500 | bZIP | cagtcacgtggc |
|  | Manes.02G046100 | MYB | CCTCCAACCTCCACA |
|  | Manes.13G107900 | MYB | tggaggttggagg |
|  | Manes.06G092600 | MYB | AACCCTCCAACCTCC |
|  | Manes.10G052500 | NAC | TGACAAGCCAAC |
|  | Manes.16G023800 | NAC | cacgtaac |
|  | Manes.07G124500 | Trihelix | tatttttttaaccgt |
|  | Manes.09G106100 | Trihelix | gtcacgtggcaatttgg |
| LOC110609679(MeF3H) | Manes.05G130800 | Nin-like | GTGGCAGCAGCAATT |
|  | Manes.18G079600 | NAC | aacgtgacggattcgaatagtt |
|  | Manes.10G052500 | NAC | atacacgccaac |
|  | Manes.15G003000 | MYB | agggggtagttaggc |
|  | Manes.14G104200 | MYB | ggggtagttaggcaa |
|  | Manes.02G046100 | MYB | TGCCTAACTACCCCC |
|  | Manes.14G011000 | MYB | CAATTTGCCTAACTACCCCCT |
|  | Manes.08G106900 | MYB | CCTAACTACCCCCTT |
|  | Manes.05G037100 | MYB | aagggggtagttagg |
|  | Manes.18G075600 | ERF | acccctaaactatcaccgcc |
|  | Manes.02G208000 | ERF | cccctaaactatcaccgcca |
|  | Manes.08G048500 | ERF | ctatcaccgcc |
|  | Manes.18G040400 | ERF | aaactatcaccgcca |
|  | Manes.13G120400 | ERF | aaactatcaccgcca |
|  | Manes.15G031500 | CPP | AATTAAAATTTAAAT |
|  | Manes.02G195200 | C2H2 | ccctcccttccacctaaac |
|  | Manes.05G151500 | BBR-BPC | ATCTACCACTCTATCTCTCTA |
|  | Manes.17G089900 | BBR-BPC | tagagagatagagtggtagataaa |
|  | Manes.17G089900 | BBR-BPC | gagagatagagtggtagataaaag |
|  | Manes.17G089900 | BBR-BPC | GCTGGGGAGAGTGGGGGAAAGTGT |
|  | Manes.05G151500 | BBR-BPC | cactttcccccactctcccca |
|  | Manes.17G089900 | BBR-BPC | gagatagagtggtagataaaagga |
|  | Manes.05G151500 | BBR-BPC | CTACCACTCTATCTCTCTACA |
|  | Manes.17G089900 | BBR-BPC | TGGGGAGAGTGGGGGAAAGTGTGG |
| LOC110612286(MeCHS1) | Manes.18G050100 | bHLH | GCCCGTTGGCACGTGAAGTGT |
|  | Manes.01G193400 | bHLH | cacacttcacgtgccaacggg |
|  | Manes.06G080300 | bHLH | cacgtgccaacggg |
|  | Manes.06G079000 | bHLH | cacgtgccaacgggc |
|  | Manes.09G181800 | bHLH | TGGCACGTGA |
|  | Manes.15G039300 | bHLH | ttcacgtgcc |
|  | Manes.15G039300 | bHLH | GGCACGTGAA |
|  | Manes.06G080300 | bHLH | cacgtgatctttag |
|  | Manes.02G195200 | C2H2 | ccatcacctcaacttcctc |
|  | Manes.02G195200 | C2H2 | ATCTAACCTCCTCCTCCAC |
|  | Manes.02G195200 | C2H2 | CCTCCTCCTCCACATTTCG |
|  | Manes.02G195200 | C2H2 | TAACCTCCTCCTCCACATT |
|  | Manes.02G195200 | C2H2 | tctccatcacctcaacttc |
|  | Manes.17G055700 | Dof | aaataggtaataaagtgaaaa |
|  | Manes.07G135300 | ERF | GGAAGTTGAGGTGATGGAGAA |
|  | Manes.06G156400 | ERF | CCTCCTCCTCCACATTTCGTG |
|  | Manes.07G135300 | ERF | AGCGGTGAGATAGATGGTGAA |
|  | Manes.09G146100 | ERF | tgtggaggaggaggt |
|  | Manes.09G146100 | ERF | AAAGGAAGCGGTGAG |
|  | Manes.08G069300 | FAR1 | acttcacgtgccaac |
|  | Manes.03G077500 | MYB | gcggcttttgtggtaggtggg |
|  | Manes.05G037100 | MYB | tttgtggtaggtggg |
|  | Manes.08G106900 | MYB | CCCACCTACCACAAA |
|  | Manes.04G074900 | MYB | tttgtggtaggtggg |
|  | Manes.15G155900 | MYB | CCCACCTACCACA |
|  | Manes.14G011000 | MYB | GTTGGCTCCCACCTACCACAA |
|  | Manes.02G046100 | MYB | CTCCCACCTACCACA |
|  | Manes.03G201700 | MYB | tggtaggtgg |
|  | Manes.01G118700 | MYB | gtggtaggtggga |
|  | Manes.14G077700 | MYB | tttgtggtaggtgggagcc |
|  | Manes.10G143200 | MYB | gtggtaggtgg |
|  | Manes.07G005800 | MYB | gtggtaggtgg |
|  | Manes.14G011000 | MYB | gcatagccttaccaaacccac |
|  | Manes.18G103600 | MYB | tggtaggtggg |
|  | Manes.18G067400 | MYB | gcttttgtggtaggtgggagc |
|  | Manes.18G067400 | MYB | AAGTGTGGGTTTGGTAAGGCT |
|  | Manes.13G107900 | MYB | ttgtggtaggtgg |
|  | Manes.06G164800 | MYB | tggtaggtgg |
|  | Manes.03G077500 | MYB | GTGAAGTGTGGGTTTGGTAAG |
|  | Manes.09G135700 | MYB | GGCTCCCACCTACCACAAA |
|  | Manes.05G205700 | MYB | ttgtggtaggtggga |
|  | Manes.02G066400 | TCP | ggtgggagccaacacgaaatgtggaggagg |
|  | Manes.12G007700 | TCP | GGCTCCCACC |
| LOC110612862(MeCHS3) | Manes.10G108100 | ARF | GCCGACAC |
|  | Manes.13G013200 | ARR-B | GCAGATACGG |
|  | Manes.18G029600 | BES1 | gcccacgtggccaaccgcct |
|  | Manes.09G181800 | bHLH | GGCCACGTGG |
|  | Manes.01G193400 | bHLH | ctcagcccacgtggccaaccg |
|  | Manes.09G181800 | bHLH | gcccacgtgg |
|  | Manes.13G043000 | bHLH | GGTTGGCCACGTGG |
|  | Manes.12G104100 | bHLH | cccacgtgg |
|  | Manes.18G050100 | bHLH | GCGGTTGGCCACGTGGGCTGA |
|  | Manes.12G104100 | bHLH | GCCACGTGG |
|  | Manes.14G112400 | bHLH | cccacgtggc |
|  | Manes.14G112400 | bHLH | GCCACGTGGG |
|  | Manes.13G043000 | bHLH | ctcagcccacgtgg |
|  | Manes.01G193400 | bHLH | GGTTGGCCACGTGGGCTGAGT |
|  | Manes.06G079000 | bHLH | cacgtggccaaccgc |
|  | Manes.06G079000 | bHLH | CACGTGGGCTGAGTG |
|  | Manes.15G039300 | bHLH | cccacgtggc |
|  | Manes.15G039300 | bHLH | GCCACGTGGG |
|  | Manes.13G136900 | bZIP | ccacgtggcc |
|  | Manes.01G249900 | bZIP | GGCCACGTGGGCTGA |
|  | Manes.08G134500 | bZIP | agcccacgtggc |
|  | Manes.15G191900 | bZIP | tcagcccacgtggcc |
|  | Manes.18G037900 | bZIP | CGGTTGGCCACGTGGGCT |
|  | Manes.09G056100 | bZIP | ctcagcccacgtggccaa |
|  | Manes.18G048100 | bZIP | tcagcccacgtggcc |
|  | Manes.08G134500 | bZIP | TGGCCACGTGGG |
|  | Manes.13G136900 | bZIP | CCACGTGGGC |
|  | Manes.05G003300 | bZIP | tcagcccacgtggcc |
|  | Manes.03G011000 | ERF | CGGCAGATACGGTGGATGG |
|  | Manes.13G143500 | ERF | GATCGGCAGATACGGTGGATG |
|  | Manes.04G094600 | ERF | ccatccaccgtatctgccgat |
|  | Manes.06G156400 | ERF | catccaccgtatctgccgatc |
|  | Manes.04G094600 | ERF | atcttcgccgttgaatttgtc |
|  | Manes.06G156400 | ERF | acccatccaccgtatctgccg |
|  | Manes.02G208000 | ERF | cccatccaccgtatctgccg |
|  | Manes.09G146100 | ERF | GGCAGATACGGTGGA |
|  | Manes.03G044100 | ERF | ATGGACGGCAGGCG |
|  | Manes.15G066800 | ERF | tctctctaatcttcgccgttg |
|  | Manes.07G135300 | ERF | AGGATCGGCAGATACGGTGGA |
|  | Manes.07G135300 | ERF | ATCGGCAGATACGGTGGATGG |
|  | Manes.01G170000 | G2-like | GGCAGATACGGTGG |
|  | Manes.05G189600 | GATA | tcttcgccgttgaat |
|  | Manes.12G000100 | LBD | acccatccaccgtatctgccg |
|  | Manes.06G108700 | LBD | ccatccaccgtatctgccgat |
|  | Manes.08G106900 | MYB | TCCAACAACCAACCC |
|  | Manes.03G077500 | MYB | ACTGTGGAGGTGTTAGGTATA |
|  | Manes.05G037100 | MYB | gggttggttgttgga |
|  | Manes.18G067400 | MYB | GTGGAGGTGTTAGGTATAGGT |
|  | Manes.06G092600 | MYB | CAACAACCAACCCAT |
|  | Manes.02G058900 | MYB | GGTGTTAGGTATAG |
|  | Manes.14G011000 | MYB | ACCTAAATCCAACAACCAACC |
|  | Manes.02G046100 | MYB | tggccaaccgcctgc |
|  | Manes.04G141700 | MYB_related | taccctagct |
|  | Manes.05G130800 | Nin-like | attgcagcagaggca |
|  | Manes.06G141800 | TCP | gaccccac |
|  | Manes.06G141800 | TCP | GACCCCAC |
|  | Manes.11G108500 | TCP | GTGGGGTCAAT |
|  | Manes.11G108500 | TCP | gtggggtcaat |
| LOC110613186(FNSI1) | Manes.13G001800 | AP2 | TATAAAGAGGGAGAGAGAGA |
|  | Manes.13G001800 | AP2 | aaaaaaaaaaaaaatagaga |
|  | Manes.13G001800 | AP2 | aaaaaaaaaaaaaaaataga |
|  | Manes.18G025700 | B3 | AAAACATTTTTGTCAGAAAAT |
|  | Manes.17G089900 | BBR-BPC | TAGATATAAAGAGGGAGAGAGAGA |
|  | Manes.17G089900 | BBR-BPC | AATAGATATAAAGAGGGAGAGAGA |
|  | Manes.05G151500 | BBR-BPC | ctctctctccctctttatatc |
|  | Manes.05G151500 | BBR-BPC | ttgagtctctctctccctctt |
|  | Manes.17G089900 | BBR-BPC | GATATAAAGAGGGAGAGAGAGACT |
|  | Manes.05G151500 | BBR-BPC | gagtctctctctccctcttta |
|  | Manes.05G151500 | BBR-BPC | ttttgagtctctctctccctc |
|  | Manes.17G089900 | BBR-BPC | TATAAAGAGGGAGAGAGAGACTCA |
|  | Manes.05G151500 | BBR-BPC | ctctctccctctttatatcta |
|  | Manes.17G089900 | BBR-BPC | AAGAGGGAGAGAGAGACTCAAAAT |
|  | Manes.17G089900 | BBR-BPC | TAAAGAGGGAGAGAGAGACTCAAA |
|  | Manes.05G151500 | BBR-BPC | ctctccctctttatatctatt |
|  | Manes.05G151500 | BBR-BPC | AACTCACTCTCTCTATTTTTT |
|  | Manes.05G151500 | BBR-BPC | CTCACTCTCTCTATTTTTTTT |
|  | Manes.05G151500 | BBR-BPC | gtctctctctccctctttata |
|  | Manes.17G089900 | BBR-BPC | aaaaaaaaaaaatagagagagtga |
|  | Manes.17G089900 | BBR-BPC | GGAATAGATATAAAGAGGGAGAGA |
|  | Manes.17G089900 | BBR-BPC | gagtagtagggagtgagaaaaata |
|  | Manes.17G089900 | BBR-BPC | aaaaaaaatagagagagtgagttc |
|  | Manes.17G089900 | BBR-BPC | aaaatagagagagtgagttcgaaa |
|  | Manes.15G130800 | GRAS | AAGAGGGAGAGAGAGACTCA |
|  | Manes.15G130800 | GRAS | GAGGGAGAGAGAGACTCAAA |
|  | Manes.05G111900 | MIKC_MADS | CCTAAAATGGAA |
|  | Manes.05G041900 | MIKC_MADS | TCTCTCTATTTTTTTTTTTTT |
|  | Manes.07G011600 | MIKC_MADS | gcttccattttaggtatga |
|  | Manes.05G041900 | MIKC_MADS | TCTCTATTTTTTTTTTTTTTT |
|  | Manes.17G062000 | MIKC_MADS | ttgcttccattttaggtat |
|  | Manes.05G041900 | MIKC_MADS | CTCTATTTTTTTTTTTTTTTT |
|  | Manes.01G263800 | MIKC_MADS | ATACCTAAAATGGAAGCAA |
|  | Manes.05G153000 | MIKC_MADS | TACCTAAAATGGAAG |
|  | Manes.13G009600 | MIKC_MADS | CCTAAAATGGAAG |
|  | Manes.05G041900 | MIKC_MADS | tttgagtctctctctccctct |
|  | Manes.05G184900 | TALE | gagtctctctctccctcttt |
|  | Manes.08G101700 | Trihelix | cggctattttgcccttaa |
|  | Manes.09G136200 | WOX | agttaattggt |
|  | Manes.03G051300 | WRKY | ttttgactttttg |
| LOC110615791(MeDFR2) | Manes.05G039400 | HD-ZIP | gcattaattga |
|  | Manes.06G092600 | MYB | CAAGCCCCACCCACA |
|  | Manes.01G011200 | NAC | cttgtgaaagacgtta |
|  | Manes.03G002200 | NAC | TAACGTCTTTCACAAGTA |
|  | Manes.05G126800 | NAC | acttgtgaaagacgttat |
|  | Manes.15G169500 | NAC | tacttgtgaaagacg |
|  | Manes.02G081200 | NAC | cttgtgaaagacgtta |
|  | Manes.03G105700 | NAC | cttgtgaaagacgttattt |
| LOC110617654(MeF3'H) | Manes.10G108100 | ARF | accgacac |
|  | Manes.13G013200 | ARR-B | TAAGATACGG |
|  | Manes.04G091800 | B3 | gcacacatgcattga |
|  | Manes.15G031500 | CPP | atttaaaatttaaaa |
|  | Manes.14G079000 | Dof | aaaatttaaaaagtaaaaa |
|  | Manes.01G245200 | ERF | CAAGGTGTCGGTGAC |
|  | Manes.05G052000 | ERF | AGGTGTCGGTGACTA |
|  | Manes.03G011000 | ERF | TGCGGTCTGCGGTGGTAGT |
|  | Manes.14G029600 | ERF | AGGTGTCGGTGAC |
|  | Manes.06G143100 | ERF | AGGTGTCGGTGAC |
|  | Manes.13G120400 | ERF | ttgtagtcaccgaca |
|  | Manes.08G048500 | ERF | ctaccaccgca |
|  | Manes.14G029500 | ERF | AGGTGTCGGTGACTA |
|  | Manes.07G135300 | ERF | ACCAGCCAAGGTGTCGGTGAC |
|  | Manes.18G040400 | ERF | ttgtagtcaccgaca |
|  | Manes.18G069000 | ERF | caccgacacc |
|  | Manes.09G146100 | ERF | GCGGTCTGCGGTGGT |
|  | Manes.08G069300 | FAR1 | TCTCAACGCGCGAGC |
|  | Manes.08G106900 | MYB | gctaactaccaccgc |
|  | Manes.02G046100 | MYB | gagctaactaccacc |
|  | Manes.03G077500 | MYB | CGGTCTGCGGTGGTAGTTAGC |
|  | Manes.01G253000 | MYB_related | agatatttttc |
| LOC110618709(MeCHI2) | Manes.18G025700 | B3 | tgaatttttttcgtgggaaat |
|  | Manes.18G025700 | B3 | CTAAGTTTTATGGAGGGAAAA |
|  | Manes.09G181800 | bHLH | AGCCACGTGA |
|  | Manes.01G269700 | bHLH | CCACGTGA |
|  | Manes.01G193400 | bHLH | GCCAAGCCACGTGATTGGCTG |
|  | Manes.12G104100 | bHLH | GCCACGTGA |
|  | Manes.06G079000 | bHLH | cacgtggcttggctc |
|  | Manes.16G015900 | bHLH | agccaatcacgtgg |
|  | Manes.13G136900 | bZIP | tcacgtggct |
|  | Manes.08G134500 | bZIP | caatcacgtggc |
|  | Manes.09G056100 | bZIP | agccaatcacgtggcttg |
|  | Manes.18G048100 | bZIP | gccaatcacgtggct |
|  | Manes.14G055900 | C2H2 | GGAAACAGAGTA |
|  | Manes.02G195200 | C2H2 | tttcctcaagcacctcccc |
|  | Manes.18G099800 | C2H2 | acctccaccaaacca |
|  | Manes.15G031500 | CPP | tattgaaattcaaaa |
|  | Manes.06G100000 | E2F/DP | gaacaatttttccctccataa |
|  | Manes.07G135300 | ERF | GGAAAATGGTTTGGTGGAGGT |
|  | Manes.07G135300 | ERF | tggtctggtggtgttggatgc |
|  | Manes.14G011000 | MYB | ctgcacctccaccaaaccatt |
|  | Manes.18G067400 | MYB | TAGTGGAAGTTGGGTGAGCTT |
|  | Manes.14G011000 | MYB | aacttccactacctaccacct |
|  | Manes.01G226200 | MYB | TTACCTACCC |
|  | Manes.03G077500 | MYB | GAGGGAAAATGGTTTGGTGGA |
|  | Manes.03G201700 | MYB | TGGTAGGTAG |
|  | Manes.02G046100 | MYB | ccactacctaccacc |
|  | Manes.14G011000 | MYB | CGCTCATTTTACCTACCCTAC |
|  | Manes.08G106900 | MYB | actacctaccacctt |
|  | Manes.18G103600 | MYB | gggtaggtaaa |
|  | Manes.01G118700 | MYB | agggtaggtaaaa |
|  | Manes.18G103600 | MYB | TGGTAGGTAGT |
|  | Manes.05G037100 | MYB | AAGGTGGTAGGTAGT |
|  | Manes.14G011000 | MYB | CTGCATAACCACCAAACCATC |
|  | Manes.13G107900 | MYB | AGGTGGTAGGTAG |
|  | Manes.04G074900 | MYB | AAATGGTTTGGTGGA |
|  | Manes.15G155900 | MYB | TTTACCTACCCTA |
|  | Manes.02G058900 | MYB | TGGGTTAGGTATAA |
|  | Manes.06G164800 | MYB | TGGTAGGTAG |
|  | Manes.02G058900 | MYB | tagggtaggtaaaa |
|  | Manes.18G067400 | MYB | acgagtagggtaggtaaaatg |
|  | Manes.01G226200 | MYB | ctacctacca |
|  | Manes.02G046100 | MYB | cacccaacttccact |
|  | Manes.05G037100 | MYB | agtagggtaggtaaa |
|  | Manes.15G003000 | MYB | TTTGGTGGGTTAGGT |
|  | Manes.01G118700 | MYB | TGGTTTGGTGGAG |
| LOC110619630(MeCHI1) | Manes.13G001800 | AP2 | aagaaggaggaggagaaaag |
|  | Manes.13G001800 | AP2 | aaggagaaggaggagaaagg |
|  | Manes.13G001800 | AP2 | aggagaaaaggaagaagagg |
|  | Manes.13G001800 | AP2 | aggagaaaaggaagaagagg |
|  | Manes.13G001800 | AP2 | gggaaaaggagaaggagaag |
|  | Manes.17G089900 | BBR-BPC | gggaaaaggagaaggagaagaaga |
|  | Manes.17G089900 | BBR-BPC | aggaggaggagaaggagaaggagg |
|  | Manes.17G089900 | BBR-BPC | aggagaaggagaagaagaagaaga |
|  | Manes.17G089900 | BBR-BPC | aggaggaggagaaaaggaagaaga |
|  | Manes.17G089900 | BBR-BPC | gaggaggagaaaaggaagaagagg |
|  | Manes.17G089900 | BBR-BPC | aggagaagaagaagaagaaggagg |
|  | Manes.05G151500 | BBR-BPC | CTCCTTCTCCTTCTCCTCCTC |
|  | Manes.17G089900 | BBR-BPC | gagaaggagaaggaggagaaagga |
|  | Manes.17G089900 | BBR-BPC | gaggaggagaaggagaaggaggag |
|  | Manes.17G089900 | BBR-BPC | ggaggaggaggagaaggagaagga |
|  | Manes.17G089900 | BBR-BPC | aggagaaggagaaggaggagaaag |
|  | Manes.17G089900 | BBR-BPC | atcaggaggaggaggagaaggaga |
|  | Manes.02G195200 | C2H2 | CCTCATCCTCCTCCTCCTC |
|  | Manes.02G195200 | C2H2 | CCTCATCCTCATCCTCCTC |
|  | Manes.02G195200 | C2H2 | TCTCCTTCTCCTCCTCCTC |
|  | Manes.02G195200 | C2H2 | CATCCTCATCCTCCTCCTC |
|  | Manes.02G195200 | C2H2 | CCTTCTCCTTCTCCTCCTC |
|  | Manes.02G195200 | C2H2 | CCTCCTTCTCCTTCTCCTC |
|  | Manes.02G195200 | C2H2 | CCTTCTCCTCCTCCTCCTG |
|  | Manes.02G195200 | C2H2 | CATCCTCCTCCTCCTCCTG |
|  | Manes.02G195200 | C2H2 | TCTCCTCATCCTCATCCTC |
|  | Manes.02G195200 | C2H2 | CCTCCTCCTCCTGATCCTC |
|  | Manes.02G195200 | C2H2 | TCTCCTCCTTCTCCTTCTC |
|  | Manes.02G195200 | C2H2 | CCTCCTCCTGATCCTCTTC |
|  | Manes.02G195200 | C2H2 | TCTCCTCCTCCTCCTGATC |
|  | Manes.02G195200 | C2H2 | CTTTCTCCTCCTTCTCCTT |
|  | Manes.02G195200 | C2H2 | TTTTCTCCTCATCCTCATC |
|  | Manes.02G195200 | C2H2 | CCTCCTGATCCTCTTCTTC |
|  | Manes.02G195200 | C2H2 | TTTTCTCCTCATCCTTCTC |
|  | Manes.02G195200 | C2H2 | CCTCCTCCTCCTCCTGTGT |
|  | Manes.02G195200 | C2H2 | TCTCCTCCTCCTTCTTCTT |
|  | Manes.02G195200 | C2H2 | CCTCCTCCTTCTTCTTCTT |
|  | Manes.02G195200 | C2H2 | TTTTCTCCTCCTCCTTCTT |
|  | Manes.02G195200 | C2H2 | TCCTTTTCTCCTCATCCTC |
|  | Manes.02G195200 | C2H2 | TCTTCTCCTTCTCCTTTTC |
|  | Manes.02G195200 | C2H2 | TCTTCTTCTTCTCCTTCTC |
|  | Manes.02G195200 | C2H2 | ATCCTTTCTCCTCCTTCTC |
|  | Manes.02G195200 | C2H2 | CTCCTTCTTCTTCTTCTTC |
|  | Manes.02G195200 | C2H2 | CCTCATCCTTCTCATTCTT |
|  | Manes.02G195200 | C2H2 | TCCTTTTCTCCTCCTCCTT |
|  | Manes.02G195200 | C2H2 | TCTTCCTTTTCTCCTCCTC |
|  | Manes.02G195200 | C2H2 | TCTCCTCATCCTTCTCATT |
|  | Manes.02G195200 | C2H2 | TCTTCTTCTCCTTCTCCTT |
|  | Manes.02G195200 | C2H2 | CCTCTTCTTCCTTTTCTCC |
|  | Manes.02G195200 | C2H2 | CCTCTTCTTCCTTTTCTCC |
|  | Manes.02G195200 | C2H2 | CCTTCTTCTTCTTCTTCTC |
|  | Manes.02G195200 | C2H2 | CCTGATCCTCTTCTTCCTT |
|  | Manes.02G195200 | C2H2 | CTTCTTCTTCTTCTTCTCC |
|  | Manes.02G195200 | C2H2 | CTTCTTCTTCTTCTCCTTC |
|  | Manes.02G195200 | C2H2 | CTCCTCCTTCTTCTTCTTC |
|  | Manes.02G195200 | C2H2 | CCTCCTTCTTCTTCTTCTT |
|  | Manes.02G195200 | C2H2 | CCCGATCCTCTTCTTCCTT |
|  | Manes.02G195200 | C2H2 | CCTTCTCCTTTTCCCGATC |
|  | Manes.02G195200 | C2H2 | CTCCTCCTCCTTCTTCTTC |
|  | Manes.02G195200 | C2H2 | TTTCCCGATCCTCTTCTTC |
|  | Manes.02G195200 | C2H2 | CATCCTTCTCATTCTTTTC |
|  | Manes.02G195200 | C2H2 | TCTTCCTTTTCTCCTCATC |
|  | Manes.02G195200 | C2H2 | CTTCTTCTTCTCCTTCTCC |
|  | Manes.02G195200 | C2H2 | GATCCTCTTCTTCCTTTTC |
|  | Manes.02G195200 | C2H2 | GATCCTCTTCTTCCTTTTC |
|  | Manes.02G195200 | C2H2 | CTATTTTCTCCTCATCCTT |
|  | Manes.02G195200 | C2H2 | TCTCCTTCTCCTTTTCCCG |
|  | Manes.02G195200 | C2H2 | CCGATCCTTTCTCCTCCTT |
|  | Manes.15G031500 | CPP | ATTTAAAATTTAAAT |
|  | Manes.03G174900 | CPP | CCAAATTTTAAAA |
|  | Manes.12G095300 | Dof | CTTCTTCTCCTTCTCCTTTTC |
|  | Manes.11G131600 | Dof | TTCTTCTCCTTCTCCTTTTCC |
|  | Manes.07G115400 | Dof | CTTCCTTTTCTCCTCCTCCTT |
|  | Manes.12G095300 | Dof | CCGATCCTCTTCTTCCTTTTC |
|  | Manes.14G065600 | Dof | aaggatcgggaaaaggagaag |
|  | Manes.12G095300 | Dof | CTGATCCTCTTCTTCCTTTTC |
|  | Manes.14G065600 | Dof | ggaggaggagaaaaggaagaa |
|  | Manes.12G095300 | Dof | AACCTTCTCCATCAACTTTTC |
|  | Manes.07G135300 | ERF | ggaggagaaggagaaggagga |
|  | Manes.07G135300 | ERF | ggaggaggaggagaaggagaa |
|  | Manes.02G208000 | ERF | CTCCTTCTCCTCCTCCTCCT |
|  | Manes.06G156400 | ERF | CCTCATCCTCCTCCTCCTCCT |
|  | Manes.02G208000 | ERF | CTCATCCTCCTCCTCCTCCT |
|  | Manes.06G156400 | ERF | CATCCTCCTCCTCCTCCTGTG |
|  | Manes.15G066800 | ERF | CCTCATCCTCCTCCTCCTCCT |
|  | Manes.07G135300 | ERF | ggaggaggaggatgaggatga |
|  | Manes.07G135300 | ERF | ggagaaggagaaggaggagaa |
|  | Manes.07G135300 | ERF | agaggatcaggaggaggagga |
|  | Manes.15G066800 | ERF | TCTCCTTCTCCTCCTCCTCCT |
|  | Manes.07G135300 | ERF | ggatcaggaggaggaggagaa |
|  | Manes.15G066800 | ERF | CCTTCTCCTTCTCCTCCTCCT |
|  | Manes.02G208000 | ERF | CTCCTTCTCCTTCTCCTCCT |
|  | Manes.06G156400 | ERF | TCTCCTCCTCCTCCTGATCCT |
|  | Manes.02G208000 | ERF | CTTCTCCTTCTCCTCCTCCT |
|  | Manes.09G146100 | ERF | ggaggaggaggagga |
|  | Manes.13G143500 | ERF | aggaggaggaggaggatgagg |
|  | Manes.07G135300 | ERF | aacacaggaggaggaggagga |
|  | Manes.06G156400 | ERF | CCTCCTCCTCCTCCTGTGTTG |
|  | Manes.06G156400 | ERF | TCTCCTCCTTCTCCTTCTCCT |
|  | Manes.07G135300 | ERF | acaggaggaggaggaggatga |
|  | Manes.03G011000 | ERF | cacaggaggaggaggagga |
|  | Manes.07G135300 | ERF | ggaggatgaggatgaggagaa |
|  | Manes.06G156400 | ERF | CCTTCTCCTTCTCCTCCTCCT |
|  | Manes.01G085200 | ERF | CCTCCTCCTCCTCCT |
|  | Manes.02G208000 | ERF | ATCCTCATCCTCCTCCTCCT |
|  | Manes.13G143500 | ERF | caacacaggaggaggaggagg |
|  | Manes.03G011000 | ERF | aggaggaggaggaggatga |
|  | Manes.15G066800 | ERF | CATCCTCATCCTCCTCCTCCT |
|  | Manes.07G135300 | ERF | agaagaggatcaggaggagga |
|  | Manes.06G156400 | ERF | CCTCATCCTCATCCTCCTCCT |
|  | Manes.06G156400 | ERF | CCTCCTCCTCCTGATCCTCTT |
|  | Manes.13G143500 | ERF | aggagaaggagaaggaggaga |
|  | Manes.15G066800 | ERF | CCTCCTTCTCCTTCTCCTCCT |
|  | Manes.07G135300 | ERF | gaagaagaagaaggaggagga |
|  | Manes.15G066800 | ERF | CCTCATCCTCATCCTCCTCCT |
|  | Manes.13G143500 | ERF | aggatcaggaggaggaggaga |
|  | Manes.13G143500 | ERF | cacaggaggaggaggaggatg |
|  | Manes.13G143500 | ERF | aggaggaggagaaggagaagg |
|  | Manes.02G208000 | ERF | CTCATCCTCATCCTCCTCCT |
|  | Manes.02G208000 | ERF | CTCCTCCTTCTCCTTCTCCT |
|  | Manes.07G135300 | ERF | gaagaagaaggaggaggagaa |
|  | Manes.03G011000 | ERF | aggatcaggaggaggagga |
|  | Manes.13G143500 | ERF | aagaggatcaggaggaggagg |
|  | Manes.03G011000 | ERF | aggaggaggaggagaagga |
|  | Manes.13G143500 | ERF | aggaggaggatgaggatgagg |
|  | Manes.07G135300 | ERF | ggaggaggaggaggatgagga |
|  | Manes.06G156400 | ERF | TCTCCTCCTCCTTCTTCTTCT |
|  | Manes.02G208000 | ERF | CTCCTCATCCTCATCCTCCT |
|  | Manes.13G143500 | ERF | aggaggaggaggagaaggaga |
|  | Manes.13G143500 | ERF | aggaggagaaggagaaggagg |
|  | Manes.03G011000 | ERF | aggaggaggaggatgagga |
|  | Manes.01G085200 | ERF | CCTTCTCCTCCTCCT |
|  | Manes.01G085200 | ERF | TCTCCTCCTCCTCCT |
|  | Manes.09G146100 | ERF | ggaggaggaggagaa |
|  | Manes.04G094600 | ERF | TTCTCCTCCTTCTCCTTCTCC |
|  | Manes.06G156400 | ERF | CCTTCTCCTCCTCCTCCTGAT |
|  | Manes.04G094600 | ERF | TCCTCCTCCTCCTGATCCTCT |
|  | Manes.13G143500 | ERF | agaagaagaaggaggaggaga |
|  | Manes.04G094600 | ERF | TCCTTCTCCTTCTCCTCCTCC |
|  | Manes.06G156400 | ERF | CCTCCTCCTTCTTCTTCTTCT |
|  | Manes.04G094600 | ERF | TTCTCCTCCTCCTCCTGATCC |
|  | Manes.01G085200 | ERF | CATCCTCCTCCTCCT |
|  | Manes.07G041300 | GATA | CCTCATCCTCATCCTCCTC |
|  | Manes.07G041300 | GATA | CCTGATCCTCTTCTTCCTT |
|  | Manes.15G130800 | GRAS | aggaggaggagaaaaggaag |
|  | Manes.15G130800 | GRAS | gggaaaaggagaaggagaag |
|  | Manes.15G130800 | GRAS | aggatgaggagaaaaggaag |
|  | Manes.15G130800 | GRAS | aggagaaggagaagaagaag |
|  | Manes.15G130800 | GRAS | aggagaaggagaaggaggag |
|  | Manes.15G130800 | GRAS | aggaggaggagaaggagaag |
|  | Manes.15G130800 | GRAS | gagaagaagaagaagaagga |
|  | Manes.15G130800 | GRAS | aagaagaagaagaaggagga |
|  | Manes.06G108700 | LBD | TTCTCCTTCTCCTCCTCCTCC |
|  | Manes.06G108700 | LBD | TCCTCATCCTCCTCCTCCTCC |
|  | Manes.06G108700 | LBD | TCCTCCTCCTCCTCCTGTGTT |
|  | Manes.06G108700 | LBD | TCCTCCTTCTCCTTCTCCTCC |
|  | Manes.06G108700 | LBD | TCATCCTCATCCTCCTCCTCC |
|  | Manes.06G108700 | LBD | TCCTTCTCCTTCTCCTCCTCC |
|  | Manes.06G108700 | LBD | TCATCCTCCTCCTCCTCCTGT |
|  | Manes.12G000100 | LBD | CATCCTCCTCCTCCTCCTGTG |
|  | Manes.06G108700 | LBD | TCCTTCTCCTCCTCCTCCTGA |
|  | Manes.06G108700 | LBD | TCCTCATCCTCATCCTCCTCC |
|  | Manes.12G000100 | LBD | CCTCATCCTCCTCCTCCTCCT |
|  | Manes.12G000100 | LBD | CCTTCTCCTTCTCCTCCTCCT |
|  | Manes.06G108700 | LBD | TTCTCCTCCTCCTCCTGATCC |
|  | Manes.06G108700 | LBD | TTCTCCTCCTTCTCCTTCTCC |
|  | Manes.06G108700 | LBD | TTCTCCTCATCCTCATCCTCC |
|  | Manes.12G000100 | LBD | CCTCATCCTCATCCTCCTCCT |
|  | Manes.12G000100 | LBD | TCTCCTCCTTCTCCTTCTCCT |
|  | Manes.06G108700 | LBD | TCCTCCTCCTCCTGATCCTCT |
|  | Manes.06G108700 | LBD | TCCTCCTCCTGATCCTCTTCT |
|  | Manes.12G000100 | LBD | CCTTCTCCTCCTCCTCCTGAT |
|  | Manes.06G108700 | LBD | TCCTCCTCCTTCTTCTTCTTC |
|  | Manes.12G000100 | LBD | CCTCCTCCTCCTGATCCTCTT |
|  | Manes.05G041900 | MIKC_MADS | CTTCTTCTTCTCCTTCTCCTT |
|  | Manes.05G041900 | MIKC_MADS | CTTCTCCTTCTCCTTTTCCCG |
|  | Manes.05G041900 | MIKC_MADS | CTTCTTCTTCTTCTTCTCCTT |
|  | Manes.05G041900 | MIKC_MADS | CTCCTCCTTCTCCTTCTCCTC |
|  | Manes.05G041900 | MIKC_MADS | CTCCTCCTTCTTCTTCTTCTT |
|  | Manes.05G041900 | MIKC_MADS | CTCCTTCTTCTTCTTCTTCTC |
|  | Manes.05G041900 | MIKC_MADS | CCTCTTCTTCCTTTTCTCCTC |
|  | Manes.05G041900 | MIKC_MADS | CCTCTTCTTCCTTTTCTCCTC |
|  | Manes.05G041900 | MIKC_MADS | CCTCCTTCTCCTTCTCCTCCT |
|  | Manes.05G041900 | MIKC_MADS | TCTCCTCCTCCTTCTTCTTCT |
|  | Manes.05G041900 | MIKC_MADS | TCTCCTTCTCCTCCTCCTCCT |
|  | Manes.05G041900 | MIKC_MADS | CCTCCTTCTTCTTCTTCTTCT |
|  | Manes.05G041900 | MIKC_MADS | CTTTCTCCTCCTTCTCCTTCT |
|  | Manes.05G041900 | MIKC_MADS | TCTTCTTCTTCTTCTCCTTCT |
|  | Manes.05G041900 | MIKC_MADS | TCTTCTTCTCCTTCTCCTTTT |
|  | Manes.05G041900 | MIKC_MADS | CCTCCTCCTTCTTCTTCTTCT |
|  | Manes.05G041900 | MIKC_MADS | TCCTTTTCTCCTCCTCCTTCT |
|  | Manes.05G041900 | MIKC_MADS | CTTCTCCTTTTCCCGATCCTT |
|  | Manes.05G041900 | MIKC_MADS | TCTTCCTTTTCTCCTCCTCCT |
|  | Manes.05G041900 | MIKC_MADS | TCTTCTCCTTCTCCTTTTCCC |
|  | Manes.05G041900 | MIKC_MADS | CCTTCTTCTTCTTCTTCTCCT |
|  | Manes.05G041900 | MIKC_MADS | TTTTCTCCTCCTCCTTCTTCT |
|  | Manes.05G041900 | MIKC_MADS | CCTCATCCTCCTCCTCCTCCT |
|  | Manes.05G041900 | MIKC_MADS | TCTCCTCCTTCTCCTTCTCCT |
|  | Manes.05G041900 | MIKC_MADS | CCTTCTCCTTCTCCTCCTCCT |
|  | Manes.05G041900 | MIKC_MADS | TCTTCTTCTTCTCCTTCTCCT |
|  | Manes.05G139200 | MYB | CTCATCCTCATCCTC |
|  | Manes.08G089700 | MYB | gaagaagaggatcaggaggag |
|  | Manes.13G107900 | MYB | AGAGGGTAGGTTA |
|  | Manes.10G018000 | MYB | CTCCTCATCCTCAT |
|  | Manes.07G125100 | MYB | CTCCTCATCCTCAT |
|  | Manes.13G107900 | MYB | GCGTGGTAGCTGG |
|  | Manes.06G092600 | MYB | GTACTACCAACTGCC |
|  | Manes.18G067400 | MYB | tggagaaggttagttgaagat |
|  | Manes.02G046100 | MYB | CTACCAACTGCCCAT |
|  | Manes.05G184900 | TALE | CCTTCTCCTTCTCCTCCTCC |
|  | Manes.05G184900 | TALE | ctctcccttctcccttgtcg |
|  | Manes.05G184900 | TALE | CATCCTCCTCCTCCTCCTGT |
|  | Manes.05G184900 | TALE | TCTCCTTCTCCTCCTCCTCC |
|  | Manes.05G184900 | TALE | TTTTCTCCTCCTCCTTCTTC |
|  | Manes.05G184900 | TALE | TCTCCTCCTTCTCCTTCTCC |
|  | Manes.05G184900 | TALE | CTTTCTCCTCCTTCTCCTTC |
|  | Manes.05G184900 | TALE | CCTCATCCTCCTCCTCCTCC |
|  | Manes.05G184900 | TALE | TCTTCCTTTTCTCCTCCTCC |
|  | Manes.05G184900 | TALE | TTCCCGATCCTTTCTCCTCC |
|  | Manes.05G184900 | TALE | CCTCCTTCTCCTTCTCCTCC |
|  | Manes.05G184900 | TALE | CATCCTCATCCTCCTCCTCC |
|  | Manes.05G184900 | TALE | TCCTTTTCTCCTCCTCCTTC |
|  | Manes.05G184900 | TALE | TCTTCTTCTTCTCCTTCTCC |
|  | Manes.05G184900 | TALE | TTCTCCTTCTCCTCCTCCTC |
|  | Manes.05G184900 | TALE | TCCTCCTCCTCCTCCTGTGT |
| LOC110625883(MeCHS2) | Manes.13G001800 | AP2 | ataaaaaaaaaaaagaaaaa |
|  | Manes.14G013300 | bHLH | GGTACCACTTGCAT |
|  | Manes.02G197900 | bHLH | tgcaagtggta |
|  | Manes.12G104100 | bHLH | cccgcgtgg |
|  | Manes.08G047200 | bHLH | TACCACTTGCA |
|  | Manes.13G136900 | bZIP | ccgcgtggcc |
|  | Manes.15G139200 | C2H2 | aaataaagacaaaac |
|  | Manes.S010100 | C2H2 | aaaataaagacaaaaca |
|  | Manes.04G140300 | C2H2 | aaaataaagacaaaacaa |
|  | Manes.12G086200 | C2H2 | aaaataaagacaaaacaatc |
|  | Manes.18G099800 | C2H2 | TCCTCCTCCGGGTGA |
|  | Manes.12G134300 | CAMTA | ccgcgtggc |
|  | Manes.06G012800 | CAMTA | ggcccgcgt |
|  | Manes.07G115400 | Dof | TTTTCTTTTTTTTTTTTATTT |
|  | Manes.12G095300 | Dof | ctccctttaattttccttttt |
|  | Manes.11G131600 | Dof | tccctttaattttcctttttt |
|  | Manes.03G044100 | ERF | ATGGACGGAGGGTC |
|  | Manes.05G041900 | MIKC_MADS | TTCTTTTTTTTTTTTATTTTG |
|  | Manes.07G011600 | MIKC_MADS | cttcccttgtttggaaaga |
|  | Manes.14G077700 | MYB | GGGAAGGTGGTTGGTTAGA |
|  | Manes.09G135700 | MYB | tctaaccaaccaccttccc |
|  | Manes.02G046100 | MYB | taaccaaccaccttc |
|  | Manes.08G106900 | MYB | accaaccaccttccc |
|  | Manes.05G037100 | MYB | AAGGTGGTTGGTTAG |
|  | Manes.13G107900 | MYB | AGGTGGTTGGTTA |
|  | Manes.18G067400 | MYB | GGGAAGGTGGTTGGTTAGATG |
|  | Manes.13G107900 | MYB | GGAGGGTCGATGG |
|  | Manes.14G039400 | MYB_related | AAATTGGATAGGATT |
| LOC110625897(MeFNSI2) | Manes.02G195200 | C2H2 | cttacatctccacctcatc |
|  | Manes.02G195200 | C2H2 | tctccacctcatctactac |
|  | Manes.02G195200 | C2H2 | tctcttacatctccacctc |
|  | Manes.05G049500 | C2H2 | GGTAGTAG |
|  | Manes.07G135300 | ERF | GGTAGTAGATGAGGTGGAGAT |
|  | Manes.13G143500 | ERF | GGGTAGTAGATGAGGTGGAGA |
|  | Manes.06G156400 | ERF | tctccacctcatctactaccc |
|  | Manes.07G135300 | ERF | GTGGGTAGTAGATGAGGTGGA |
|  | Manes.13G107400 | HD-ZIP | cctaatcatt |
|  | Manes.13G009600 | MIKC_MADS | ccacaaatgggaa |
|  | Manes.18G103600 | MYB | gggtaggtaga |
|  | Manes.03G201700 | MYB | gggtaggtag |
|  | Manes.06G164800 | MYB | gggtaggtag |
|  | Manes.15G155900 | MYB | TCTACCTACCCCC |
|  | Manes.05G037100 | MYB | atgggggtaggtaga |
|  | Manes.14G011000 | MYB | AGAAAGATCTACCTACCCCCA |
|  | Manes.08G106900 | MYB | TCTACCTACCCCCAT |
|  | Manes.13G107900 | MYB | tgggggtaggtag |
|  | Manes.03G077500 | MYB | tgcaaaatgggggtaggtaga |
|  | Manes.06G092600 | MYB | catctactacccact |
|  | Manes.01G226200 | MYB | CTACCTACCC |
|  | Manes.04G074900 | MYB | atgggggtaggtaga |
|  | Manes.14G077700 | MYB | GAGAGGTTGGGTGAGAGCT |
| LOC110628286(MeANS) | Manes.13G001800 | AP2 | AGGAAAAAAAAAACAAGAAA |
|  | Manes.13G001800 | AP2 | AAGAAAAAAAGGAAAAAAAA |
|  | Manes.13G001800 | AP2 | GAAAAAAAGGAAAAAAAAAA |
|  | Manes.13G001800 | AP2 | GAAGAAAAAAAGGAAAAAAA |
|  | Manes.13G001800 | AP2 | AAAAACAAGAAAAAGAGAGT |
|  | Manes.13G001800 | AP2 | AGAAAAAAAGGAAAAAAAAA |
|  | Manes.13G001800 | AP2 | AAAAGGAAAAAAAAAACAAG |
|  | Manes.17G089900 | BBR-BPC | AATGGAGAGTGATGGAGAAGGGAG |
|  | Manes.06G080300 | bHLH | cacgtgacaactga |
|  | Manes.05G003300 | bZIP | taggctgacgtgaca |
|  | Manes.08G134500 | bZIP | ggctgacgtgac |
|  | Manes.16G120300 | bZIP | taggctgacgtgaca |
|  | Manes.04G004100 | bZIP | gctgacgtgac |
|  | Manes.09G031700 | bZIP | taggctgacgtgaca |
|  | Manes.12G140100 | bZIP | taggctgacgtgaca |
|  | Manes.07G095100 | C2H2 | GAGTGATGGAG |
|  | Manes.02G195200 | C2H2 | cctttttttcttcatcttc |
|  | Manes.11G131600 | Dof | ttgttttttttttcctttttt |
|  | Manes.12G095300 | Dof | cttgttttttttttccttttt |
|  | Manes.14G065600 | Dof | GATGAAGAAAAAAAGGAAAAA |
|  | Manes.07G115400 | Dof | ctctctttttcttgttttttt |
|  | Manes.07G115400 | Dof | tttcctttttttcttcatctt |
|  | Manes.17G055700 | Dof | GATGAAGAAAAAAAGGAAAAA |
|  | Manes.07G115400 | Dof | ttttttttttcctttttttct |
|  | Manes.07G115400 | Dof | tttttttttcctttttttctt |
|  | Manes.03G039500 | GRF | ACAAGTGTGTCAGA |
|  | Manes.03G039500 | GRF | ACAAGTGTGTCAGA |
|  | Manes.05G041900 | MIKC_MADS | tgttttttttttccttttttt |
|  | Manes.05G041900 | MIKC_MADS | ttttttttttcctttttttct |
|  | Manes.01G025700 | MIKC_MADS | CCATCCAAATATAGCAAG |
|  | Manes.05G041900 | MIKC_MADS | tcttgttttttttttcctttt |
|  | Manes.05G041900 | MIKC_MADS | ttttcttgttttttttttcct |
|  | Manes.05G041900 | MIKC_MADS | gttttttttttcctttttttc |
|  | Manes.05G041900 | MIKC_MADS | tttttttttcctttttttctt |
|  | Manes.05G041900 | MIKC_MADS | ctttttcttgttttttttttc |
|  | Manes.05G041900 | MIKC_MADS | tctttttcttgtttttttttt |
|  | Manes.05G041900 | MIKC_MADS | ttttttcctttttttcttcat |
|  | Manes.05G153000 | MIKC_MADS | ATCCAAATATAGCAA |
|  | Manes.05G041900 | MIKC_MADS | ttcttgttttttttttccttt |
|  | Manes.05G041900 | MIKC_MADS | tttcttgttttttttttcctt |
|  | Manes.10G143200 | MYB | GGTGTTGGTGG |
|  | Manes.07G005800 | MYB | GGTGTTGGTGG |
|  | Manes.13G107900 | MYB | TGGGTGTTGGTGG |
|  | Manes.06G092600 | MYB | ctaccaccaacaccc |
|  | Manes.06G092600 | MYB | ccaccaacacccaca |
|  | Manes.10G143200 | MYB | GTGGGTGTTGG |
|  | Manes.07G005800 | MYB | GTGGGTGTTGG |
|  | Manes.14G011000 | MYB | taaagctaccaccaacaccca |
|  | Manes.14G011000 | MYB | CCCTGCTCTCCCCTACCCATT |
|  | Manes.03G028900 | NAC | AGAGTGATGGAGAAG |
|  | Manes.13G141600 | NAC | cttctccatcactct |
|  | Manes.16G032500 | NAC | AGAGTGATGGAGAAG |
|  | Manes.01G145500 | WOX | tcagtcattca |
|  | Manes.05G056100 | WRKY | CAGTTGACCG |
|  | Manes.01G007600 | WRKY | cggtcaac |
| LOC110629198(F3'5'H) | Manes.13G001800 | AP2 | gaaggagaggaaaaaagaga |
|  | Manes.13G001800 | AP2 | aggaaggagaggaaaaaaga |
|  | Manes.13G001800 | AP2 | gagaggaaaaaagagataag |
|  | Manes.17G089900 | BBR-BPC | aggaaggagaggaaaaaagagata |
|  | Manes.06G012800 | CAMTA | aggccgcgt |
|  | Manes.12G134300 | CAMTA | CCGCGTGCA |
|  | Manes.15G031500 | CPP | ATTTAAAATTTAAAA |
|  | Manes.15G031500 | CPP | ATTTAAAATTTAAAA |
|  | Manes.04G052000 | EIL | GCCCGGGTTCAATGA |
|  | Manes.13G081100 | ERF | cggggggtttgctgcgcag |
|  | Manes.11G146600 | GATA | gcatccagatcggag |
|  | Manes.15G130800 | GRAS | aggaaggagaggaaaaaaga |
|  | Manes.13G107400 | HD-ZIP | CCTAATCATT |
|  | Manes.05G041900 | MIKC_MADS | TCTCTTTTTTCCTCTCCTTCC |
|  | Manes.05G041900 | MIKC_MADS | CTCTCTTATCTCTTTTTTCCT |
|  | Manes.01G226200 | MYB | ttacctaccc |
|  | Manes.15G155900 | MYB | tttacctaccctt |
|  | Manes.18G103600 | MYB | GGGTAGGTAAA |
|  | Manes.01G118700 | MYB | AGGGTAGGTAAAA |
|  | Manes.02G058900 | MYB | AAGGGTAGGTAAAA |
|  | Manes.04G074900 | MYB | CCAAGGGTAGGTAAA |
|  | Manes.01G144500 | NAC | cttggcatgcacg |
|  | Manes.02G055900 | TCP | GCCGGGTCCACCA |
|  | Manes.01G263300 | TCP | CCGGGTCCAC |
|  | Manes.02G066400 | TCP | GCCGGGTCCACCATGAGCCCGGGTTCAATG |
|  | Manes.12G007700 | TCP | CGGGTCCACC |
|  | Manes.18G103100 | TCP | tggtggacccggc |
|  | Manes.02G055900 | TCP | ATCGGGTCCGCGT |
|  | Manes.07G132100 | Trihelix | TCACTCCGGCGGGCT |
|  | Manes.16G040100 | Trihelix | TTCACTCCGGCGGGC |
|  |  |  |  |

| **Supplementary Table S5. The TFs that showed high Pearson correlation coefficients with flavonoid biosynthesis genes** | | | | |
| --- | --- | --- | --- | --- |
| **Target genes** | **ID** | **TFs** | **Gene family** | **PCC** |
| 110602917(MeANR) | 110606884 | Manes.18G029600 | BES1 | 0.861591655 |
|  | 110611236 | Manes.03G189400 | SBP | 0.936912311 |
|  | 110615581 | Manes.05G022000 | bHLH | 0.817767311 |
|  | 110628584 | Manes.12G108500 | MYB | 0.888941753 |
|  | 110611236 | Manes.03G189400 | SBP | 0.940430477 |
|  | 110606884 | Manes.18G029600 | BES1 | 0.9650511 |
| 110605890(MeDFR1) | 110615940 | Manes.01G263800 | MIKC_MADS | -0.946860099 |
|  | 110602590 | Manes.15G066800 | ERF | 0.827174277 |
|  | 110609504 | Manes.02G178100 | bZIP | 0.830016465 |
|  | 110629858 | Manes.13G120400 | ERF | 0.830305934 |
|  | 110616467 | Manes.06G131200 | ERF | 0.840318056 |
|  | 110621814 | Manes.08G099800 | ERF | 0.917050474 |
|  | 110610587 | Manes.03G044100 | ERF | 0.807972336 |
|  | 110629858 | Manes.13G120400 | ERF | 0.815522287 |
|  | 110602590 | Manes.15G066800 | ERF | 0.873478062 |
|  | 110605879 | Manes.18G040400 | ERF | 0.907409845 |
|  | 110609504 | Manes.02G178100 | bZIP | 0.934563737 |
| 110606120(MeFLS) | 110630416 | Manes.13G107900 | MYB | -0.867235666 |
|  | 110614813 | Manes.01G249900 | bZIP | -0.852768995 |
|  | 110616656 | Manes.06G092600 | MYB | 0.832757246 |
|  | 110622868 | Manes.09G181800 | bHLH | 0.851098317 |
|  | 110616474 | Manes.01G269700 | bHLH | 0.912653602 |
|  | 110630017 | Manes.13G136900 | bZIP | 0.917110877 |
|  | 110630249 | Manes.13G043000 | bHLH | 0.932379665 |
|  | 110614813 | Manes.01G249900 | bZIP | -0.858250573 |
|  | 110616656 | Manes.06G092600 | MYB | 0.801199185 |
|  | 110601836 | Manes.15G191900 | bZIP | 0.86212403 |
|  | 110630249 | Manes.13G043000 | bHLH | 0.911821432 |
|  | 110616474 | Manes.01G269700 | bHLH | 0.994856183 |
| 110609679(MeF3H) | 110605879 | Manes.18G040400 | ERF | 0.813019089 |
|  | 110615182 | Manes.05G130800 | Nin-like | 0.846090843 |
|  | 110605879 | Manes.18G040400 | ERF | 0.923172367 |
|  | 110615182 | Manes.05G130800 | Nin-like | 0.958048361 |
| 110612286(MeCHS1) | 110631036 | Manes.14G077700 | MYB | 0.876765415 |
| 110612862(MeCHS3) | 110627902 | Manes.12G104100 | bHLH | -0.886180472 |
|  | 110601836 | Manes.15G191900 | bZIP | 0.815990749 |
|  | 110615182 | Manes.05G130800 | Nin-like | 0.859504661 |
|  | 110602590 | Manes.15G066800 | ERF | 0.978232844 |
|  | 110630249 | Manes.13G043000 | bHLH | 0.819380647 |
|  | 110617590 | Manes.06G141800 | TCP | 0.866836837 |
|  | 110615182 | Manes.05G130800 | Nin-like | 0.940397936 |
|  | 110602590 | Manes.15G066800 | ERF | 0.961096708 |
|  | 110606884 | Manes.18G029600 | BES1 | 0.971527579 |
| 110613186(FNSI1) | 110605712 | Manes.17G089900 | BBR-BPC | -0.821316557 |
| 110617654(MeF3'H) | 110600888 | Manes.15G031500 | CPP | -0.844994238 |
|  | 110605879 | Manes.18G040400 | ERF | 0.939839099 |
|  | 110600888 | Manes.15G031500 | CPP | -0.803619596 |
|  | 110600068 | Manes.14G079000 | Dof | 0.853254864 |
|  | 110605879 | Manes.18G040400 | ERF | 0.976155127 |
| 110618709(MeCHI2) | 110627902 | Manes.12G104100 | bHLH | -0.800611584 |
|  | 110616474 | Manes.01G269700 | bHLH | 0.881468681 |
| 110619630(MeCHI1) | 110621326 | Manes.01G085200 | ERF | 0.824535611 |
|  | 110615679 | Manes.05G151500 | BBR-BPC | 0.848141794 |
|  | 110618750 | Manes.07G115400 | Dof | 0.848170323 |
|  | 110624050 | Manes.10G018000 | MYB | 0.855126899 |
| 110625883(MeCHS2) | 110627902 | Manes.12G104100 | bHLH | -0.89463113 |
|  | 110613601 | Manes.04G140300 | C2H2 | 0.920685307 |
|  | 110631036 | Manes.14G077700 | MYB | 0.889206145 |
|  | 110613601 | Manes.04G140300 | C2H2 | 0.94203055 |
|  | 110600323 | Manes.14G039400 | MYB_related | 0.949326466 |
| 110625897(MeFNSI2) | 110612998 | Manes.04G074900 | MYB | 0.824146903 |
|  | 110631036 | Manes.14G077700 | MYB | 0.861859961 |
|  | 110629037 | Manes.13G107400 | HD-ZIP | 0.935555724 |
| 110628286(MeANS) | 110631036 | Manes.14G077700 | MYB | 0.859124767 |
| 110629198(MeF3'5'H) | 110606830 | Manes.18G103100 | TCP | 0.83132597 |
|  | 110619333 | Manes.07G132100 | Trihelix | 0.85746675 |
|  | 110606830 | Manes.18G103100 | TCP | 0.877123199 |
|  | 110629037 | Manes.13G107400 | HD-ZIP | 0.916320185 |
